# Pharmacological Interventions for Bacterial Prostatitis

**DOI:** 10.3389/fphar.2020.00504

**Published:** 2020-04-30

**Authors:** Situ Xiong, Xiaoqiang Liu, Wen Deng, Zhengtao Zhou, Yulei Li, Yechao Tu, Luyao Chen, Gongxian Wang, Bin Fu

**Affiliations:** ^1^Department of Urology, The First Affiliated Hospital of Nanchang University, Nanchang, China; ^2^Jiangxi Institute of Urology, Nanchang, China

**Keywords:** bacterial prostatitis, multidrug-resistant *E. coli*, bacterial biofilms, *E. faecalis*–*Enterococcus faecalis*, Fosfomycin, phage therapy, plant extracts, combination therapy

## Abstract

Prostatitis is a common urinary tract condition but bring innumerable trouble to clinicians in treatment, as well as great financial burden to patients and the society. Bacterial prostatitis (acute bacterial prostatitis plus chronic bacterial prostatitis) accounting for approximately 20% among all prostatitis have made the urological clinics complain about the genital and urinary systems all over the world. The international challenges of antibacterial treatment (emergence of multidrug-resistant bacteria, extended-spectrum beta-lactamase-producing bacteria, bacterial biofilms production and the shift in bacterial etiology) and the transformation of therapeutic strategy for classic therapy have attracted worldwide attention. To the best of our knowledge currently, there is not a single comprehensive review, which can completely elaborate these important topics and the corresponding treatment strategy in an effective way. This review summarizes the general treatment choices for bacterial prostatitis also provides the alternative pharmacological therapies for those patients resistant or intolerant to general treatment.

## Introduction

Prostatitis is a common but bothersome urinary tract disease in urological practice that annoys patients and urologists worldwide. The incidence rate of prostatitis just follows those of prostate cancer (PCA) and benign prostate hyperplasia (BPH) in all male urinary tract conditions. Prostatitis may be the most prevalent urinary tract disease in patients below 50 years old (Collins et al., 1998; Schaeffer, 2006). According to the statistics, nearly 16% men reported a history of prostatitis at some point in their lives, and prostatitis makes up nearly 25% of urologist visits globally (Collins et al., 1998; Khan et al., 2017). The global annual cost for primary diagnosis and management is a stupendous sum (over $84 million), excluding medical expenditure and cost of the lost productivity, and appears to increase with time (Pontari et al., 2007). High cost brings invisible economic pressure to patients and the society.

The National Institutes of Health (NIH) has divided prostatitis into four categories *via* clinical characteristics, including acute bacterial prostatitis (ABP) (category I), chronic bacterial prostatitis (CBP) (category II), chronic prostatitis/chronic pelvic pain syndrome (CPPS) (category III), and asymptomatic inflammatory prostatitis (category IV) (Krieger et al., 1999). The most common among prostatitis is category III, which affects nearly 90% of patients diagnosed with prostatitis. Although the prevalence rate of bacterial prostatitis (approximately 20% of all prostatitis cases) is not the highest in the four categories, ABP carries potential risk of critical morbidity from abscess, sepsis, and septic shock, if insufficiently managed. CBP is particularly prone to relapse, which could lead to decreased libido, erectile dysfunction, and premature ejaculation, all of which may severely affect the quality of life and mental health of patients. In addition, the increasing difficulty of antibacterial treatment (emergence of multidrug-resistant bacteria and extended-spectrum beta-lactamase (ESBL)-producing *Escherichia coli*, bacterial biofilms production, and shift in bacterial etiology) and the transformation of therapeutic strategy have attracted worldwide attention.

In this article, we focused on bacterial prostatitis (categories I and II), with an emphasis on current general pharmacological therapy regimens, new international therapeutic challenges, and alternative pharmacological therapeutic strategies for patients who are resistant or intolerant to general treatment. We aimed to provide clinicians with new ideas in treating patients with complex bacterial prostatitis and offer patients the hope of overcoming the disease.

## ABP (NIH Category I)

ABP is a prostatic bacterial inflammation that causes pelvic pain, systemic symptoms (fever, chills, nausea, and vomiting), and voiding symptoms (frequency, urgency, odynuria, dysuria, and urinary retention in severe cases) (Krieger et al., 1999). A tender, swollen, and hot prostate is almost always tangible in rectal examination. The incidence peaks of ABP are in males 20–40 years old and those older than 70 years old (Roberts et al., 1998).

### Pathogenesis and Pathogenic Microorganisms

Several natural defenses against infection for the prostate gland are present, such as the production of antibacterial substances and the mechanical flushing of the prostatic urethra *via* voiding and ejaculation (Fair and Parrish, 1981). Nonetheless, bacteria still cause acute prostatitis by ascending urethral infection from the external urethral meatus, by flowing back from contaminated urine to the ejaculatory and prostate duct after transurethral manipulations (e.g., catheterization and cystoscopy), and by being implanted during a prostate biopsy directly (Millan-Rodriguez et al., 2006; Kim et al., 2014; Coker and Dierfeldt, 2016; Gill and Shoskes, 2016). Other pathogenic mechanisms include lymphatic invasion from the rectum and hematogenous infection (Ramakrishnan and Salinas, 2010). The incidence and prevalence of ABP are not completely known. However, in general, the incidence rate of ABP in the communities is three times higher than in hospitals (Etienne et al., 2008). According to the epidemiological survey of ABP, *E. coli* (accounting for 65%–80%) comprises the overwhelming majority of the bacteria that cause this infection. Other causal agents include *Enterococcus*, *Pseudomonas aeruginosa*, *Proteus*, *Klebsiella*, *Enterobacter*, and *Serratia* (Yoon et al., 2012)*. Neisseria gonorrhoeae*, *Chlamydia trachomatis*, certain fungi (*Cryptococcus*, *Salmonella*, and *Candida*), and *Mycobacterium tuberculosis* also reportedly cause ABP, especially among sexually active and immunocompromised patients (Brede and Shoskes, 2011; Nagy and Kubej, 2012; Gill and Shoskes, 2016).

### Evaluation

Serum laboratory assessment for ABP generally reveals elevated inﬂammatory markers, such as white blood cells, neutrophils, C-reactive protein, and erythrocyte sedimentation rate (Sharp et al., 2010). In a previous study, white blood cells higher than 18,000 per mm^3^ (18 * 10^9^/L) and blood urea nitrogen level higher than 19 mg/dl (6.8 mmol/L) are independently associated with severe ABP cases (Yazawa et al., 2013). Approximately 70% of patients show abnormally elevated prostate-specific antigen (PSA) caused by the inflammatory destruction of epithelial cells in the prostate ducts. However, this condition sometimes should be differentiated from PCA (Ludwig, 2008). Elevated PSA levels would decline to normal after 1–2 months of treatment; if not, PCA should be considered (Ludwig, 2008; Sharp et al., 2010; Brede and Shoskes, 2011). Routine urine tests often detect positive leukocyte count. The Meares–Stamey two-glass or four-glass test is not recommended for men with probable ABP, because prostatic massage as aggressive prostate palpation can release bacteria and inflammatory cytokines, thereby increasing the potential risk of bacteremia, and subsequently, sepsis (Coker and Dierfeldt, 2016). Less than 2% of men with ABP develop prostatic abscess. Patients who remain febrile after 36 h or whose symptoms do not improve with antibiotics should be evaluated for prostatic abscess. Non-contrast computed tomography (CT) scan, magnetic resonance imaging (MRI) of the pelvis, and transrectal prostatic ultrasonography (TRUS) are useful in identifying prostate abscess. During this time, prostate biopsy should not be performed to avoid inducing septicemia.

### Differential Diagnosis

According to the complex clinical manifestations of acute prostatitis and the results of numerous auxiliary examinations, the following diseases need to be identified: benign prostatic hypertrophy (BPH), CBP, CPPS, PCA, cystitis, acute pyelonephritis, epididymitis, and proctitis (Coker and Dierfeldt, 2016) (**Table 1**).

**Table 1:** Differential Diagnosis of Acute Bacterial Prostatitis.

Diagnosis	Distinguishing characteristics	Tests to rule out differential diagnoses
Benign prostatic hypertrophy	Obstructive voiding symptoms; enlarged, nontender prostate; negative urine culture	Inferior abdominal ultrasound and uroflowmetry
Chronic bacterial prostatitis	Recurrent UTIs with the same organism in prostatic secretions at least 3 months	Urinalysis with each episode; DRE; Meares–Stamey four-glass test or PPMT
Chronic pelvic pain syndrome	Pain attributed to the prostate with no demonstrable evidence of infection	Urinalysis and midstream urine culture; DRE
Prostate cancer	Presence of constitutional symptoms; presence of nodules on prostate examination	PSA testing; MRI; TRUS; prostate biopsy (only if prostate cancer suspected based on PSA and/or DRE results)
Acute cystitis	Irritative voiding symptoms; normal prostate examination	DRE; inferior abdominal ultrasound
Acute pyelonephritis	Chills; fever; lumbago and backache; urine sediment microscopic examination revealed the leucocytes casts	Physical examination; urine sediment microscopic examination
Epididymitis	Tenderness to palpation on affected epididymis; irritative voiding symptoms	Physical examination; US
Proctitis	Tenesmus; rectal bleeding; feeling of rectal fullness; passage of mucus through the rectum	DRE; stool routine examination; proctoscopy

UTI, urinary tract infection; PPMT, pre- and post-massage test; DRE, digital rectal examination; PSA, prostate-specific antigen; MRI, magnetic resonance imaging; US, ultrasonography.

## CBP (NIH Category II)

CBP is defined as a prolonged urinary tract infection (UTI) that lasts 3 months or longer and recurrent UTIs with persistent source of urinary tract bacterial seeding (Krieger and Egan, 1991). Most symptoms of CBP are similar to ABP but with rare fever, which may recur within weeks or months. Digital prostate palpation in patients with CBP detects tenderness, softening (“bogginess”), firm induration, or nodularity (Lipsky et al., 2010). The prevalence of CBP is very low, with only 5%–10% of all prostatitis cases suffering from this condition (Khan et al., 2017).

### Pathogenesis and Pathogenic Microorganisms

Distinct relationships are shown between ABP and CBP. Epidemiology showed that nearly 10% of patients with ABP may advance into CBP, and further 10% into CPPS (not discussed in this article) (Yoon et al., 2012). The factors that affected ABP evolve into CBP are diabetes, prior manipulation, not doing cystostomy, and urethral catheterization (Yoon et al., 2012). Biofilm-producing bacteria acquiring lasting vitality is the main reason for the characteristic persistence of infection despite appropriate antibiotic therapy. The current study showed that biofilm formation may result in the increased ability of strains that cause acute prostatitis to persist in the prostatic secretory system and lead to the recurrent UTIs characteristic of CBP (Soto et al., 2007). Studies suggested that secretory inhibitor of platelet microbicidal protein (SIPMP) production is associated with CBP although no consensus has been reached (Ivanov et al., 2008). The existence of increased oxidative stress and damage may be closely associated with the long course of CBP (Lou et al., 2006). In the past, *E. coli* was considered the main cause of CBP, but recent studies show that gram-positive bacteria, especially *Enterococcus faecalis*, have replaced *E. coli* and have been discovered as the causal agents (Bundrick et al., 2003; Cai et al., 2011; Heras-Canas et al., 2016). Furthermore, the pathogen that causes recurrent episodes of UTI is the same microbe. Among sexually active male patients with CBP, chronic mycoplasma infection generally leads to decreased fertility because of the impaired semen quality (including sperm vitality, sperm total motility, and percentage of progressively motile sperm) and a higher incidence of premature ejaculation (Cai et al., 2014; Shang et al., 2014).

### Evaluation

For the diagnosis of CBP, completing quantitative sequential bacteriological localization cultures is necessary. The Meares–Stamey four-glass test is considered as the gold standard for the diagnosis of CBP; in this test, first-voided urine (VB1), midstream urine (VB2), expressed prostatic secretions (EPS), and post-prostate massage urine (VB3) are sampled (Lipsky et al., 2010; Wagenlehner et al., 2013). Moreover, the diagnosis is based on the substantially lower leukocyte and bacterial counts in VB1 and VB2 compared with VB3 and EPS (Lipsky et al., 2010). Semen or ejaculate culture may increase the diagnostic utility of the four-glass test, but is not sufficient for diagnosis by it alone (Zegarra et al., 2008; Magri et al., 2009; Wagenlehner et al., 2013). Considering the complexity of the four-glass test, a straightforward and more convenient two-glass test is often performed in clinical practice for diagnosis. The two-glass pre- and post-massage test (PPMT), which requires clean catch urine specimen before massage and a first-stream urine after, provides similar results and is a reasonable alternative for at least initial evaluation (Nickel et al., 2006). PSA levels are elevated in <20% of men with this infection (Wise and Shteynshlyuger, 2008; Lipsky et al., 2010).

### Differential Diagnosis

Several conditions that present with similar symptoms or results of examination must be differentiated from CBP (Rees et al., 2015) (**Table 2**).

**Table 2:** Differential Diagnosis of Chronic Bacterial Prostatitis.

Diagnosis	Distinguishing characteristics	Tests to rule out differential diagnoses
Benign prostatic hypertrophy	Obstructive voiding symptoms; enlarged, nontender prostate; negative urine culture	Inferior abdominal ultrasound; uroflowmetry; urinalysis; midstream urine culture; DRE
Acute bacterial prostatitis	Acute episode of urinary tract symptoms, pelvic pain and systemic symptoms; history of prostate manipulation and/or high-risk sexual behavior; past medical history (e.g., BPH, urethral stricture, genitourinary infections, immunocompromised)	Urine culture with each episode (e.g., Meares–Stamey four-glass test or PPMT); DRE
Chronic pelvic pain syndrome	Pain attributed to the prostate with no demonstrable evidence of infection	Urinalysis and midstream urine culture
Prostate cancer	Presence of constitutional symptoms; presence of nodules on prostate examination	PSA testing; MRI; TRUS; prostate biopsy (only if prostate cancer suspected based on PSA and/or DRE results)
Chronic epididymitis	Tenderness and presence of nodules on affected epididymis palpation; discomfort in the scrotum and groin at least 3 months	Physical examination; US
Prostatic tuberculosis	Systemic symptoms of tuberculosis; past history of tuberculosis; irregular enlargement of the prostate and seminal vesicle and with tuberculous nodules to palpation	DRE; TRUS; X-ray examination; prostatic fluid and semen culture; prostate biopsy if necessary

BPH, benign prostatic hypertrophy; PPMT, pre- and post-massage test; DRE, digital rectal examination; PSA, prostate-specific antigen; MRI, magnetic resonance imaging; US, ultrasonography; TRUS, transrectal ultrasonography.

## General Treatment For ABP

**Figure 1:**
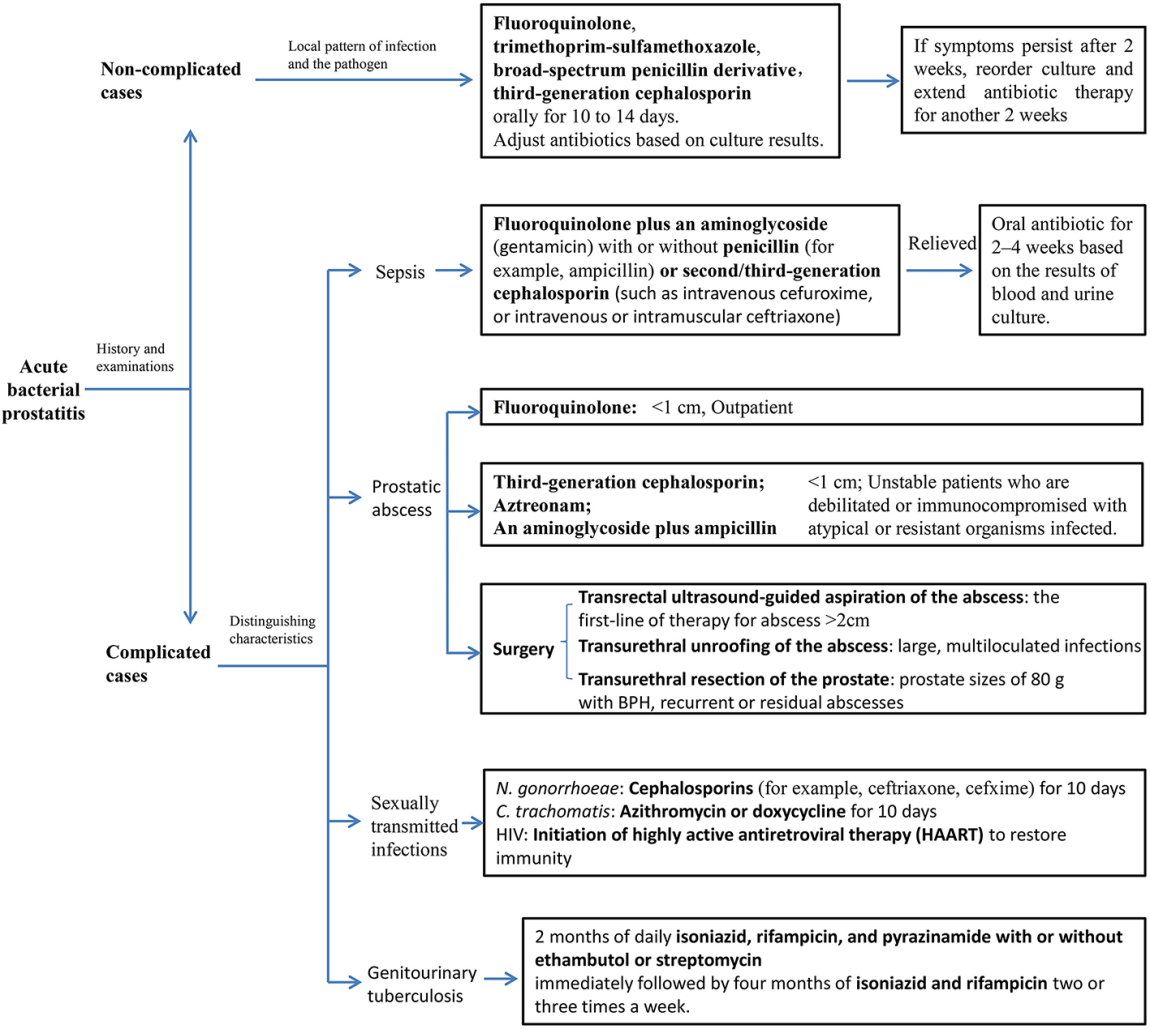
Treatment algorithm for acute bacterial prostatitis.

Successful treatment of bacterial prostatitis is based on the selection of the appropriate therapeutic method (include operation and antibiotic), which depends on the severity of symptoms, bacterial flora, local antibiotic resistance patterns, and the drug concentration in prostatic fluid (**Figure 1**).

### Classic Drug of Choice for Non-Complicated ABP

Empiric antibiotic therapy may be selected as the initial treatment according to the local epidemiology of bacterial prostatitis to presume the mode of infection and the suspected pathogen before obtaining the results of blood and urine cultures and drug sensitivity. During acute prostatic infection with inflammation, antibiotics (except nitrofurantoin) easily penetrate the prostatic tissue to achieve the sufficient effective bactericidal concentration (Nickel et al., 1995). The majority of ABP patients receive satisfactory therapeutic effect initially at outpatient service with less than 6% requiring hospitalization (Millan-Rodriguez et al., 2006). For non-severe patients without systemically ill and urinary retention, oral fluoroquinolone, trimethoprim-sulfamethoxazole, broad-spectrum penicillin derivative, or third-generation cephalosporin is usually the first choice for empirical therapy as an outpatient. The quinolones reach three to four times higher intraprostatic concentrations than β-lactam antibiotics (Goto et al., 1998).

### Classic Drug Choice for Complicated ABP

An acutely ill patient with a clinical picture of sepsis or a systemic inflammatory response syndrome must be accepted for hospitalization with parenteral antibiotics that broadly cover fluoroquinolone plus an aminoglycoside or a combination of the above with a penicillin or second/third-generation cephalosporin (Brede and Shoskes, 2011). Once the clinical condition of the patient is stable without fever and urinary retention, oral antibiotic therapy should replace intravenous medication for 2–4 weeks, based on the results of blood and urine culture and drug sensitivity. It is necessary to plan repeat urine culture during and 1 week after therapy to ensure bacterial eradication.

Sexually active men younger than 35 years old and men older than 35 years old with high-risk sexual behavior suspected of *N. gonorrhoeae* and *C. trachomatis* infection should receive a standard treatment with cephalosporins and azithromycin or doxycycline, respectively (Skerk et al., 2004). The treatment of ABP associated with genitourinary tuberculosis is recommended by the WHO with 2 months of daily isoniazid, rifampicin, and pyrazinamide with or without ethambutol or streptomycin and immediately followed by four months of treatment with isoniazid and rifampicin two or three times a week. For complicated cases of genitourinary tuberculosis with HIV or AIDS, antituberculosis therapy should be extended to 9–12 months (Cek et al., 2005). In addition, treatment for HIV patients is based on initiation of highly active antiretroviral therapy (HAART) to restore immunity (Brede and Shoskes, 2011).

ABP associated with transrectal prostate biopsy is becoming more common with an incidence rate in the range 0.6%–2.1% (Stoica et al., 2007; Miura et al., 2008; Shigehara et al., 2008; Brede and Shoskes, 2011). Also, fluoroquinolone-resistant strains of *E. coli* were observed in most cases (Stoica et al., 2007; Miura et al., 2008; Shigehara et al., 2008). A retrospective study by Shigehara et al. showed that a risk factor of fluoroquinolone-resistant *E. coli* may have been caused by the use of levofloxacin for prophylactic treatment, and to avoid this strain from generating, patients should receive levofloxacin for a short period before biopsy (Shigehara et al., 2008). Moreover, it is recommended for patients with acute prostatitis to receive treatment with cephalosporin or carbapenem after prostate biopsy (Shigehara et al., 2008). A randomized controlled trial conducted among 100 volunteers who had an indication for prostate biopsy determined the efficacy of rectum sterilization before TRUS-guided prostate biopsy to decrease bacteremia rate and sepsis complications (Kanjanawongdeengam et al., 2009).

If acute prostatitis resolves slowly or fail to respond within 36 h with continued systemic symptoms, and a fluctuant mass in the prostate is observed, we should consider the presence of prostatic abscess. Prostatic abscess, a serious complication of ABP, is particularly susceptible in patients who are immunocompromised, diabetic, with cirrhosis or renal impairment, and who are managed with an indwelling urethral catheter or clean intermittent self-catheterization (Aravantinos et al., 2008; Benway and Moon, 2008). Using CT, MRI, or TRUS to diagnosis prostate abscess is helpful. Treatment plans should be formulated according to the size of prostate abscess. Generally, drainage or conservative treatment is effective and feasible. Prostate abscesses less than 1 cm in diameter could be treated conservatively with oral drug. Although no standard of care exists, quinolones (such as ciprofloxacin) are recommended for outpatient and a third-generation cephalosporin, aztreonam, or the combination of an aminoglycoside with ampicillin for those are debilitated or immunocompromised with atypical or-resistant organisms infected (Ackerman et al., 2018). Whereas, larger ones require single aspiration or continuous drainage (Chou et al., 2004). Transrectal ultrasound-guided aspiration of the abscess is the first-line of therapy for patient with abscess larger than 2 cm with severe LUTS and/or leukocytosis (Vyas et al., 2013). If the abscess is large and with multiloculated infections, transurethral unroofing of prostate abscess might be more appropriate (Goyal et al., 2013). Besides, transurethral unroofing with resection of the prostate is fit for patient with BPH (prostate sizes of ≥80 g), recurrent or residual abscesses and persistent lower urinary tract symptoms (LUTS) (Goyal et al., 2013).

## General Treatment for CBP

**Figure 2:**
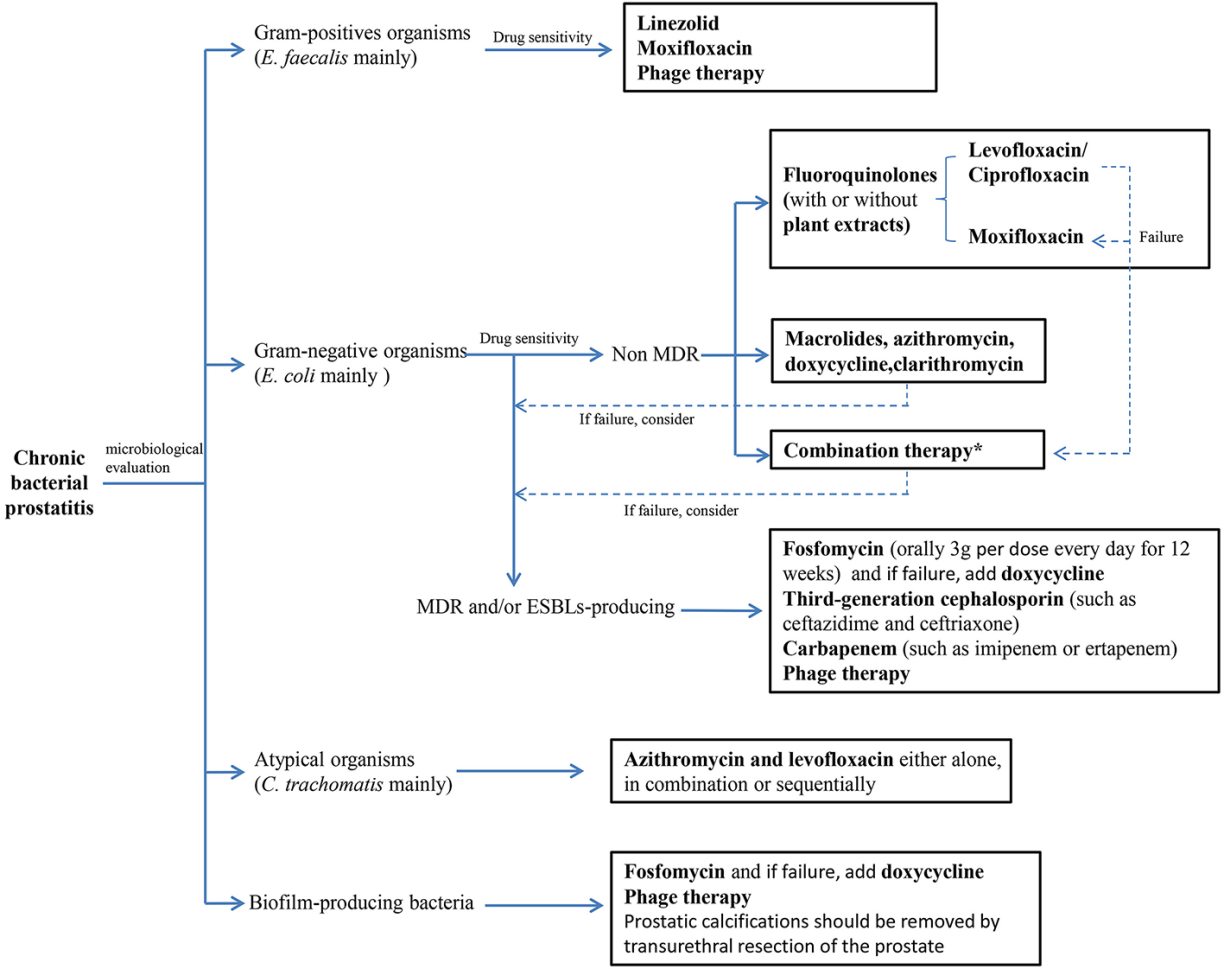
Treatment algorithm for chromic bacterial prostatitis. (MDR, multi-drug resistance; ESBLs, extended spectrum β-lactamases). *Combination therapy: Plant extracts combinate with antibiotics, antibiotics combinate with antibiotics and other drug combinate with antibiotics.

Treating CBP is challenging, because only few oral antibiotics could penetrate the prostate and achieve sufficient effective bactericidal concentration at the field of infection (Lipsky et al., 2010; Karaiskos et al., 2019). A reproducible rat model of CBP has shown that the acinar and interstitial spaces are the most susceptible part of infection (Nickel et al., 1991). Thus, pathogens at prostatic secretion and tissues must be exposed to a sufficiently high concentration of antibiotics for effective therapy that would inhibit bacterial growth or eradicate pathogens from the site of infection (Wagenlehner et al., 2005). In addition, with the emergence of multidrug resistance (MDR) and/or ESBLs-producing bacteria, biofilm-producing bacteria and the shift in bacterial etiology, the therapeutic schemes of CBP become complex and tricky (**Figure 2**).

### Mechanism of Antimicrobial Therapy for CBP

Different from ABP, the therapeutic strategy for CBP is to select antibiotics with strong ability to permeate prostatic tissue and advantageous pharmacokinetics. Drug penetration is supposedly associated with the passive transport mechanism that constitutes diffusion and concentration (Wagenlehner et al., 2005). Therapeutic agents with high pKa, low protein binding, a low degree of ionization (biological membranes do not allow the passage of charged substances), high lipid solubility, and small water-soluble molecules (small water-soluble molecules can cross biological membranes as part of free water diffusion) are recommended to treat CBP, as these factors facilitate the drug’s passage through the prostatic epithelium and penetrate into the prostatic infective tissue. The presence of a pH gradient across a biological membrane introduces the phenomenon of ion trapping. In a stable system, the uncharged fraction of a lipid-soluble drug equilibrates on the two sides of the membrane, but the charged fraction is greater on one side or the other, depending on the pH values. The greatest drug concentration (sum of charged and uncharged fractions) is on the side with the higher degree of ionization (Wagenlehner et al., 2005). The pH value of human prostatic secretion of normal men is alkaline, and the pH of prostatic secretion from men with prostatic infection is markedly increased (mean pH 8.34) (Naber and Sorgel, 2003). Thus, the concentrations of trimethoprim, a weak base with a pKa of 7.4, may be inadequate in an alkaline milieu, such as in infected prostatic secretion and seminal fluid. The fluoroquinolones in clinical use are amphoteric (neither pure acids nor bases, but have characteristics of both) or zwitterionic drugs with two ionizing groups (positively and negatively charged); thus, they have two pKa values (Sorgel et al., 2001). The amount of charged drug is minimal (isoelectric point) at a pH value between the two pKa values, whereas it is maximal at higher and lower pH values. Moreover, drugs are transferred to the side with a higher degree of ionization. Hence, some fluoroquinolones with an isoelectric point close to plasma pH may be concentrated in fluids with a pH value above and below plasma pH, such as the alkaline prostatic secretion from men with bacterial prostatitis and seminal fluid (Naber et al., 1993; Naber et al., 2001). Therefore, the concentration of some fluoroquinolones in the alkaline seminal fluid may even exceed those in the plasma. In addition, macrolides also easily penetrate into prostatic and seminal fluids.

### Classic Antibiotic Choice for CBP

A high proportion of *E. faecalis* (gram-positive bacteria) and most *E. coli* (gram-negative bacteria) have been isolated in prostatic fluid and urine of patients with CBP in a series of studies, which emphasize the necessity of selecting antibacterial agents with broad-spectrum activity. Fluoroquinolones have been internationally considered as the cornerstone for the treatment of CBP because of their broad-spectrum antimicrobial properties and advantageous pharmacokinetics in prostatic tissue. The overall rate of clinical and microbiological responses in prostatitis with CBP is 70%–90% at the end of therapy with oral fluoroquinolones, but only 60% after half a year (Khan et al., 2017). In general, for *E. coli* and other family members, such as *Enterobacteriaceae* without antibiotic resistance, bacterial eradication rate of fluoroquinolones is satisfactory in the case of CBP (Khan et al., 2017). Some studies showed that levofloxacin 500 mg once daily per os for 28 days and ciprofloxacin 500 mg twice daily for 28 days are both clinically and microbiologically effective in the treatment of CBP caused by susceptible pathogens without severe complications (Bundrick et al., 2003; Naber et al., 2008). Kurzer, E. et al. established a model that compared 90 days of double strength trimethoprim-sulfamethoxazole with 14, 28, and 60 days of ciprofloxacin at 500 mg, which indicated that ciprofloxacin 500 mg twice daily for 28 days is the most cost-effective treatment for the disease (Kurzer and Kaplan, 2002). To investigate the safety and efficacy of levofloxacin compared with ciprofloxacin in Chinese patients with CBP, a trial comparing the effects of oral levofloxacin (500 mg q.d.) and ciprofloxacin (500 mg b.i.d.) administered for 4 weeks on 408 male patients showed that levofloxacin have some advantages over ciprofloxacin in terms of the bacterial clearance rate, clinical efficacy, adverse events, and disease recurrence (Zhang et al., 2012). A study investigating the penetration of moxifloxacin into prostatic tissue in patients with benign prostatic hyperplasia showed that the concentrations of moxifloxacin in the serum and prostatic tissue were well above the minimal inhibitory concentration (MIC) values of most important prostatic pathogens after 400 mg of moxifloxacin intravenous infusion. The high tissue/serum ratio and the extended antibacterial spectrum imply that the active concentration in the prostate may translate into increased efficacy in the treatment of CBP (Wagenlehner et al., 2006). Moreover, an evidence of the effectiveness of moxifloxacin in the established pharmacokinetic (PK) model by K, Hurtado F et al., in which moxifloxacin with enhanced tissue penetration and high area under the concentration–time curve in tissue (AUC_tissue_)/MIC ratios was suggested as a better alternative to levofloxacin for the treatment of CBP when the pathogenic agent is resistant to levofloxacin (Hurtado et al., 2014). Unfortunately, the recurrence rate of CBP ranged from 25% to 50%, even with the appropriate type and duration of treatment (Weidner et al., 1999; Bundrick et al., 2003). If the source of prostatic infection cannot be eliminated, the long-term use of low-dose oral antibiotics, such as trimethoprim, for inhibitory treatment can reduce symptom recurrence, but future studies should be designed to demonstrate this viewpoint (Perletti et al., 2013).

### Other Choice for CBP

Non-steroidal anti-inflammatory drugs significantly reduce prostate-related pain (Pontari, 2002). In addition, the use of alpha-1-adrenergic antagonists contributes to prostate-related LUTS (Nickel and Touma, 2012). Experiments suggest that finasteride may prevent CBP progression, although no consensus was reached on its mechanism (Lee et al., 2011). The existence of prostate calculi or calcification is associated with the persistence of prostatic infection because of biofilm infection and is likely to relapse after antibacterial treatment (Zhao et al., 2012; Bartoletti et al., 2014). Therefore, if antibacterial treatment for the abovementioned situations failed, then the surgical removal of these factors may have positive implications for prostatitis treatment.

Acute urinary retention may occur in approximately 10% patients with ABP, including acute episodes of CBP. Urinary tract decompression is the key to remove infection and alleviate pain. The best intervention approach is intermittent or short-term indwelling urethral catheter to relieve obstruction, but this may increase the risk of ABP’s progression to CBP (Yoon et al., 2012). For patients who cannot tolerate catheters, suprapubic cystostomy may be an option. Alpha-blocker treatment is also recommended, but the clinical evidence for this approach is weak (Nickel et al., 2006).

## New International Challenges in Treatment

### MDR and/or ESBLs-Producing Bacteria

For 25 years, oral fluoroquinolones have been considered as the cornerstone of treatment for bacterial prostatitis because of their broad-spectrum bactericidal activity, which covers common pathogens associated with prostatitis and advantageous pharmacokinetics in prostatic tissue (Lipsky et al., 2010; Videcnik et al., 2015). Oral fluoroquinolones have become a major contributor to use and resistance of antibiotics because of the high incidence rate of UTIs (Costelloe et al., 2010; Zalmanovici et al., 2010). Although 10% of men diagnosed with prostatitis have bacterial infection, approximately 1/2 are treated with antibiotic therapy. The consequence of resistance due to widespread use has led to therapeutic failures with antibiotics. Fluoroquinolone resistance among clinical isolates of *E. coli* has been reported since 2002. A steep increase in urinary and prostatic infections due to MDR gram-negative *Enterobacteriaceae* globally, even in the community, and the prevalence of ESBL-producing bacteria render invalidity to fluoroquinolones and affect the choice of treatment options (de la Rosette et al., 1993; Zowawi et al., 2015; Zhanel et al., 2018). Fluoroquinolones resistance in urinary *E. coli* is reportedly as high as 70%, and approximately 60% of strains produce ESBLs simultaneously in many Asian countries, including China, India, and Vietnam (Zowawi et al., 2015). The international research community has deemed these as the greatest and most urgent universal risk (Laxminarayan et al., 2016).

MDR is defined as non-susceptibility to at least one agent in three or more antimicrobial categories (Magiorakos et al., 2012). Recent studies indicate that the increase of resistance to fluoroquinolone is due to the emergence and proliferation of a MDR subclone of sequence type 131 (ST131). ST131 also contributes to the transmission of ESBL-producing *E. coli* that mainly carry CTX-M-14 and CTXM-15 (Karlowsky et al., 2011; Denisuik et al., 2013; Karlowsky et al., 2014).

In the 1980s, the emergence of ESBLs in *Enterobacteriaceae* was witnessed globally (Zowawi et al., 2015). ESBLs can hydrolyze third-generation cephalosporins, such as ceftriaxone or ceftazidime, and aztreonam (Paterson and Bonomo, 2005). Typically, ESBLs are derived from genes for TEM-1, TEM-2, or SHV-1 by mutations that alter the amino acid configuration around the active site of these β-lactamases (Paterson and Bonomo, 2005). Presently, ESBL-producing *E. coli* and *Klebsiella pneumoniae* are prevalent in medical institutions, even in the community (Doi et al., 2013). The CTX-M-type ESBL is the most common ESBL associated with uropathogenic *Enterobacteriaceae* that may lead to global spread. ESBL-producing *E. coli* emerging in Europe and North America accounts for approximately 97% producing CTX-M-type ESBL (Hoban et al., 2012). Considering the combination with ESBL genes and other antimicrobial resistance genes on plasmids (mobile genetic elements that spread easily between bacteria), strains obtain MDR.

If the empirical treatment is ineffective, then the treatment strategy should be adjusted based on the results of bacterial culture and drug sensitivity. Third-generation cephalosporin (such as ceftazidime and ceftriaxone) and even carbapenem (such as imipenem or ertapenem) are usually alternative antibiotics for fluoroquinolone resistance (Shigehara et al., 2008). The availability of empirical treatment of combination with abroad-spectrum β-lactam drug (either a penicillin or a cephalosporin) and an aminoglycoside for patients, who are severely ill or who have recently received antibiotic therapy, have been confirmed. Carbapenems resistant to hydrolysis by ESBLs are the most frequently recommended antibiotics for the treatment of MDR bacteria (Paterson and Bonomo, 2005). However, the deeply increased expenditure of carbapenems over the past two decades around the world, particularly in developing countries, indicates the rapidly developing phenomenon of carbapenem resistance in key gram-negative pathogens (Rogers et al., 2014; Van Boeckel et al., 2014). Carbapenem resistance tends to emerge in areas where ESBL prevalence is high, driven by selection pressure from carbapenem use, and subsequently spread through international travel and globalization. The production of carbapenemases, such as KPC-type (*K. pneumoniae* carbapenemase), NDM-type (New Delhi metallo-β-lactamase), and OXA-48-type enzymes, is the significant mechanism underlying carbapenem resistance among *Enterobacteriaceae* (Poirel et al., 2012; Munoz-Price et al., 2013; Wailan and Paterson, 2014). High-level expression of ESBL or AmpC β-lactamases in conjunction with outer-membrane porin changes and increased activity of efflux pumps are other mechanisms that result in carbapenem resistance (Peleg and Hooper, 2010). The Centers for Disease Control and Prevention (CDC) have defined carbapenem-resistant *Enterobacteriaceae* as an urgent antibiotic resistance threat (Centers for Disease Control and Prevention, 2013). In addition, the infection of carbapenem-resistant *Enterobacteriaceae* leads to the death of approximately 50% of patients. Clinicians should consider local drug resistance patterns in selecting antibiotics, especially with the emergence of ESBL-producing strains in complicated UTIs, and should adjust therapy based on culture results.

### The Shift in Bacterial Etiology

As early as 2003, Bundrick et al, have described that *E. faecalis* and *E. coli* were the most common isolates; the former was the major infection among cohorts of patients with CBP (Bundrick et al., 2003). An epidemiological investigation of the uropathogens among 6221 Italian patients with CBP from January 1997 to December 2008, showed that incidence of gram-positive was higher than gram-negative bacteria, and *E. faecalis* prevalence increased within 2006–2008 (Cai et al., 2011). Moreover, *E. faecalis* prevalence has also been reported in North America (Bundrick et al., 2003; Nickel and Xiang, 2008) not just to Italy. Recently, a clinical and microbiological survey of 332 cases of CBP showed that *E. faecalis* was the main etiologic agent (37.7%), followed by *E. coli* (22.2%) among patients diagnosed microbiologically with CBP (Heras-Canas et al., 2016). These results indicate that the predominant etiologic determinant of CBP is gradually transferring to *E. faecalis*, which belongs to gram-positive microorganisms. Wagenlehner indicated that the improved clinical use of fluoroquinolones for CBP patients increased the trend of gram-positive infections (Wagenlehner et al., 2008).

The concentrations of most fluoroquinolones in the serum and prostatic tissue (eg, norfloxacin, 0.08 mg/L; ciprofloxacin, 0.18 mg/L; ciprofloxacin, 1–4 mg/L; levofloxacin, 1.42 mg/L, normalized to a 400 mg oral dose of moxifloxacin) are often below or sometimes just above the MICs for susceptible *E. faecalis* (Hallgren et al., 2001; Magri et al., 2011a). This phenomenon of changing disease epidemiology explains the relatively low eradication rates of *E. faecalis* (50%–70% with fluoroquinolones) compared with *E. coli* (between 70% and 90%) (Magri et al., 2011a). This shift in bacterial etiology has profound influence on the therapy of CBP. Unfortunately, as for oral agents, rare studies have reported so far the successful outcome of linezolid or moxifloxacin therapy which perhaps the only available fluoroquinolone that may attain prostatic secretions concentrations 10-fold above the MIC for *E. faecalis* (Hallgren et al., 2001; Wagenlehner et al., 2008). Moreover, the increase in resistance of *Enterococcus* strains secreting pathogenicity factors is more alarming, due to which chronic inflammation is maintained in the genitourinary system (Neimark et al., 2010; Stamatiou and Pierris, 2017).

The evaluation of microbiological characteristic for each CBP patients to perform a correct, appropriate, and personalized treatment schedule is essential (Cai et al., 2011). Therefore, Magri, V et al. proposed to dismiss empirical therapy to avoid catastrophic consequences in terms of chemoresistance and poor clinical practice (Magri et al., 2011a).

### Bacterial Biofilm

In addition to the production of MDR gram-negative *E. coli* and the transformation of dominant pathogenic bacteria, the formation of biofilm is also an important factor that affects the treatment of bacterial prostatitis according to urologists. Biofilms are currently defined as structured bacterial communities embedded in a self-produced exopolysaccharide matrix adherent on any abiotic or biological surface (Costerton et al., 1995). Before pathogenic microorganism form biofilms, antibiotics are the most effective treatment for ABP when bacteria are in the free-floating state. However, once biofilms are formed, the effectivity rate of antibiotic treatment is immensely decreased. Biofilms can protect bacteria from the killing activity of host-defense mechanisms and antibiotics by producing dense extracellular matrix and creating an environment in which bacteria cooperate and interact, as well as from the clearing out effect of the hydrodynamic force (Andrea et al., 2003). Hemolysin and the expression of type 1 fimbriae are related to biofilm production, which causes acute prostatitis (Soto et al., 2007). Furthermore, biofilm-producing bacteria acquiring lasting vitality are the main reason for the characteristic persistence of infection despite appropriate antibiotic therapy (Soto et al., 2007).

The current study showed that biofilm formation may result in the increased ability of strains that cause acute prostatitis to persist in the prostatic secretory system and lead to the recurrent UTIs characteristic of CBP (Soto et al., 2007). Inside the biofilms, a small fraction of cells are dormant bacteria that are almost immune to the effects of antibiotics, and these dormant inert bacteria could lead to recurrences (Kanamaru et al., 2006; Soto et al., 2007; Mazzoli, 2010). Therefore, there are significant negative influences on the clinical efficacy for antibiotic therapy to biofilm-producing bacteria. The improvement in clinical symptoms after treatment seem more associated with the decrease in bacterial biofilm production than the negative microbiological tests (Bartoletti et al., 2014). However, the survival of biofilm-producing bacteria may have caused the persistence of symptoms in patients with CBP despite apparent negative microbiological tests.

The etiology theoretically means that the whole range of bacterial species that cause prostatitis are able to form biofilms, including *E. coli*, *Staphylococcus*, *Enterococcus*, and *Ureaplasma* spp (Garcia-Castillo et al., 2008). Soto et al. suggested that biofilm formation in prostate tissue by persistent and antibiotic-resistant *E. coli* strains causes the relapse of CBP (Soto et al., 2007). The majority of the *E. coli*, *E. faecalis*, and *Staphylococcus* (85% of all strains) are strong or medium producers around the world. Only 6% of *E. coli* strains and approximately 10% of all other gram-negative strains are not biofilm producers (Bartoletti et al., 2014). Strong microorganisms that form microbial biofilm structures are often not eradicated by antibiotic treatment. Moreover, the relevance between bacterial biofilms and antibiotic-resistant *E. coli* strains that cause the relapse of symptoms has been confirmed (Cai et al., 2018).

Biofilm production increases the rate of incidence, recurrence, and drug resistance of bacterial prostatitis and threatens the ability of urologists to treat bacterial prostatitis. New therapeutic strategies should be added to eradicate bacterial biofilm formation to improve existing treatment of patients with bacterial prostatitis. Soto et al. thought the detection of biofilm-producing bacteria could be used to determine whether patients should require prolonged- or short-therapeutic regimens (Soto et al., 2007). Wu et al. suggested that the inhibition of bacterial attachment to a urothelial surface could be a crucial procedure to avoid biofilm formation (Wu et al., 1996). Future studies should be designed to explore whether effective eradication of the bacterial biofilm could be associated with a good medium- and long-term clinical outcome of treatment.

## Latest Therapeutic Strategy

### The Revival of Fosfomycin

Considering that the evolving changes in resistance rates for fluoroquinolones have a serious impact on treatment, alternative antibiotic therapies are urgently needed. Studies have demonstrated fosfomycin has a strong killing effect *in vitro* against antimicrobial-resistant *E. coli* (including ESBL-producing, AmpC-producing, and MDR isolates). This new discovery inspires the treatment of refractory bacterial prostatitis (Michalopoulos et al., 2011; Gardiner et al., 2014; Falagas and Rafailidis, 2015). Fosfomycin, an old drug used before for therapy of females uncomplicated cystitis and transrectal prostate biopsy prophylaxis, has been recently rediscovered as a treatment for MDR infections with an effective rate >90% in lowering UTIs (Pullukcu et al., 2007; Senol et al., 2010). In addition, fosfomycin-susceptibility rate of ESBL-producing *E. coli*, ESBL-producing *K. pneumoniae*, and *E. faecalis* reported lately by a systemic review was 95%, 83.8%, and 96.8%, respectively (Fan et al., 2018). Clinically, a case report of the successful administration of oral fosfomycin on patients with CBP who were infected by a complicated vancomycin-resistant *Enterococci* demonstrated that fosfomycin is available (Shrestha et al., 2000).

Fosfomycin belongs to a broad-spectrum antimicrobial class with bactericidal activity against gram-negative and gram-positive bacteria and is not relevant to any clinically approved antibiotics on the structure (Sastry and Doi, 2016). The unique bactericidal action of fosfomycin involves obstructing the synthesis of bacterial cell wall by inhibiting pyruvyl transferase, a cytoplasmic enzyme that catalyzes the first step of peptidoglycan biosynthesis (Cunha et al., 2015; Fan et al., 2018). A study involving a rat model has shown the effects of reduction of bacterial reproduction, inhibition of inflammation (with significant lower IL-6, IL-8, anti-TNF-α, and PSA levels in the prostate tissue), and improvement of prostatic tissue injury in the treatment of bacterial prostatitis using fosfomycin (Fan et al., 2018). The serum half-life (t1/2) of fosfomycin is 5.7 h, bioavailability is approximately 37%, and excretion rate through the kidneys is 60%. Fosfomycin with a large volume of distribution (Vd of ~2 L/kg) indicates extensive tissue/cellular penetration (Cunha et al., 2015). The concentration of fosfomycin can achieve therapeutic concentration (prostate levels >4 µg/g) in infected prostate and is higher than in healthy tissue (Gardiner et al., 2014; Karaiskos et al., 2019). The pharmacological properties of fosfomycin (including the high lipid solubility, small molecular size, and low protein binding) are conducive to the penetration of the parenchyma of the lipid-rich prostate (Lipsky et al., 2010; Cunha et al., 2015; Zhanel et al., 2016). Based on the present studies, fosfomycin can effectively prevent the relapse of UTIs in patients with prior history of urinary colonization and infections caused by complicated pathogens, such as ESBL-producing *E. coli* and MDR bacteria (Almeida et al., 2019). In addition, fosfomycin also has a significant effect on biofilm-producing bacteria, including *E. coli* (Corvec et al., 2013).

To determine the safety of the treatment with fosfomycin, a prospective observational study was conducted in 44 men with CBP; results showed that oral fosfomycin was well tolerated with minor side effects, even when the treatment was prolonged for 90 days (Karaiskos et al., 2019). Moreover, the study indicated that the only adverse effect was diarrhea in 18% of the participants (8 of 44). However, this effect subsided when dose intervals and/or modificatory dietary were lengthened during the course of fosfomycin administration (Karaiskos et al., 2019).

Oral fosfomycin, as an alternative therapeutic option in the case of bacterial resistance to classic antibiotic or poor tolerability to the first-line agent, is recommended at least 6 weeks up to 12 weeks (Karaiskos et al., 2019). The present evidence supports the idea that the most effective dose interval is 48 h. An oral fosfomycin dosage of 3g.q24h for the first week followed by 3g.q48h for the remaining duration appears to have the highest clinical cure rates that also minimizes gastrointestinal adverse effects (Zhanel et al., 2018). High-dose fosfomycin (> 3 g per dose) has not demonstrated enhanced clinical efficacy compared with 3 g doses of treatment (Cunha et al., 2015). Appropriate adjustment of fosfomycin dosage or frequency when adverse gastrointestinal effects occur would easily solve the problem. The presence of prostate stones and biofilm-producing bacteria contributes to the persistence of infection and easily leads to recurrence following antimicrobial therapy. Thus, the prolongation of treatment duration to a median period of 12 weeks with single oral dose of 3 g of fosfomycin has been suggested to apply without significant adverse event to the aforementioned cases (Zhao et al., 2012; Bartoletti et al., 2014). In addition, the prostatic calcifications are suggested to be removed by transurethral resection of the prostate (Cunha et al., 2015). Rhodes et al. suggested that 3 g of oral fosfomycin should be administered 1–4 h prior to prostate biopsy to prevent postoperative infection (Rhodes et al., 2015). A case of persistent ESBL-positive *E. coli* CBP refractory to antibiotic therapy showed that the combination with doxycycline and fosfomycin might have synergistic effect, in which the antibacterial activity of one antibiotic enhances the intracellular transport and/or antimicrobial activity of the other. Thus, the ability of prostate penetration and/or penetrating into his infected prostate calcifications/biofilm increased (Cunha et al., 2015).

### Phage Therapy in Bacterial Prostatitis

Phages, bacterial viruses, were used in the treatment of bacterial infections because they show characteristics of infecting and lysing bacteria that have been discovered almost a century ago. Although they were abandoned by the western world after the discovery of antibiotics, the phenomenon of the global effective decline in antibiotic therapy has forced scientists to look for alternative strategies for prophylaxis and control of bacterial infection. Phage therapy (PT) may be one of the most popular choices nowadays. Several major advantages, such as host-specificity, self-amplification, biofilm degradation, and low toxicity to humans, are attributed to PT in comparison with antibiotics therapy (Donlan, 2009; Bourdin et al., 2014). Moreover, phages are able to selectively infect and kill bacterial cells, even those that have acquired resistance to antibiotics, such as vancomycin-resistant *Enterococcus faecium* (Biswas et al., 2002).

Phages are simple but genetically diverse, non-living biological entities that mostly consist of double-stranded (ds)DNA, single-stranded (ss)DNA, ssRNA, or dsRNA (Hatfull, 2008). Phages are naturally occurring bacterial parasites that cannot generate independently, that is, they are non-living. Thus, these parasites depend on the bacterial host for survival. Phage life cycles can be divided into lytic and lysogenic period, besides they can replicate in the host bacteria at both period (Hanlon, 2007). Conventional PT relies on strictly lytic phages that obligately kill the bacterial host. Phages typically bind to specific receptors on the surface of bacterial cell, inject their genetic material into the host cell, and then either integrate this material into the bacterial genome to reproduce vertically from mother to daughter cell or hijack the bacterial replication machinery to produce the next generation of phage progeny. Upon reaching a critical mass, phage progeny can have a population of a few to over 1000 viral particles, depending on environmental factors. Environmental stressors on the bacterial host can induce the lysogenic phage from the latent prophage form, thereby triggering a transition to the lytic cycle. The latter becomes active and hydrolyzes the peptidoglycan cell wall, thereby releasing novel phage to reinitiate the lytic cycle (Weinbauer, 2004). In contrast to lytic phages, lysogenic phages integrate their genetic material into the bacterial chromosome in the form of an endogenous prophage. The ability of the phages to kill the bacterial cells forms the basis of the idea of using phages as therapeutic agents. In addition, lytic phages are compiled into preparations called “phage cocktails,” which consist of multiple phages proven to have *in vitro* efficacy against the target pathogen (Lin et al., 2017).

Given the absolute difference between phages and those of all antibiotics in terms of mechanism of action, phages are effective against multidrug-resistant bacteria (Hanlon, 2007). Phages can target pathogens without notably affecting the normal flora of bacteria because of the narrower antibacterial spectrum of phages than antibiotics (Chibani-Chennoufi et al., 2004). Similar to antibiotics, resistance mechanisms can be evolved by bacteria to resist the killing infection of lytic phages. However, the phage can also enable opposite mechanisms to avoid, circumvent, or subvert host limitations by recognizing new or altered receptors and anti-CRISPR genes (Hyman and Abedon, 2010; Labrie et al., 2010). CRISPR/Cas is programmed to disrupt antibiotic resistance genes and destroy antibiotic resistance plasmids (Yosef et al., 2015). Innovations in the gene editing tool CRISPR/Cas have created novel opportunities for PT. Interestingly, Levin et al. found that the bacterial mutant generation that resist phage might be accompanied by the decline of bacterial virulence (Levin and Bull, 2004).

Direct antibacterial action and immunomodulating effects mediated by the phages themselves and by bacterial antigens are present in the phage lysates that are used for PT (Letkiewicz et al., 2010b). Moreover, all forms of prostatitis, especially CBP, may benefit from the immunomodulating effects of phages. Phages downregulate the expressions of TLR4 and MHC class, both of which are implicated in the immunopathology of prostatitis (Van Belleghem et al., 2017). Experiments of Miedzybrodzki et al. showed that neutrophils that were stimulated both by live bacteria and by their endotoxins could induce the inhibition of the formation of reactive oxygen species in PT, even independent of the phages’ capability to hydrolyze the bacterial-peptidoglycan cell wall (Miedzybrodzki et al., 2008). This activity may play an important role in reducing the oxidative stress that accompanies the chronic-inflammatory process in the CBP (Zhou et al., 2006). Result of a significant decline in C-reactive protein level in patients treated with phage preparations suggested the anti-inflammatory properties (Miedzybrodzki et al., 2009).

Published experimental finding of Nishikawa et al. demonstrated the large potential of phage (T-even-related phage may be the suitable candidate) in the treatment of *E. coli*-induced UTIs (Nishikawa et al., 2008). A cohort of 27 patients with CBP received PT, Letkiewicz et al. reported that a significant decrease in the EPS leukocyte count, conspicuous reduction of the prostate volume, and an increase in Q_max_ were noted. As confirmed by two consecutive EPS cultures, eradication of pathogen was observed in 13 patients, without significant side effects. All these results indicated that PT could be efficient in patients with CBP (Letkiewicz et al., 2010a). The same author also reported a case of PT-eradicating *E. faecalis* in CBP (Letkiewicz et al., 2009). Encouraging results were obtained by the application of PT to bacterial eradication, such as improvement in NIH-chronic prostatitis symptom index (NIH-CPSI) and lack of early disease recurrence.

Bacterial biofilm polysaccharide normally protects the bacteria against the majority of antibiotics because of its resistance to antimicrobial treatment and removal by the host immune system. However, phages specific to *Enterobacter agglomerans* can produce the specific polysaccharide depolymerases that may be able to degrade the extracellular polysaccharide matrix of biofilms (Hughes et al., 1998). Some studies showed that phage T4 is capable of infecting and multiplying within biofilm-producing *E. coli* cells and then disrupting the morphology of the biofilm. Thus, bacterial cells are not protected from the extracellular matrix of the *E. coli* biofilms (Doolittle et al., 1995). In addition, T4-like phages (the majority of therapeutic phages belong to this family) and some antibiotics such as β-lactams, quinolones, and mitomycin C show a phenomenon of related effect that is named phage-antibiotic synergy (Comeau et al., 2007). Bacteriophage that express a biofilm-degrading enzyme during infection was engineered by Lu and Collins to attack the bacterial cells in the biofilm and to attack simultaneously the biofilm matrix composed by extracellular polymeric substances, thereby demonstrating that it is also feasible to construct genetically engineered enzymatic phages with greater efficacy against biofilm (Lu and Collins, 2007).

Recent data indicated that phages are capable of penetrating the epithelial cell layers and spreading throughout the cell structure and subsequently the body, including the blood, lymph, organs, and even the brain (Nguyen et al., 2017). Furthermore, Gorski et al. developed a phage bank that was armed with organ-specific peptides, which enable them to home in on target organs, including prostate, and assure efficient and stable eradication of infections (Gorski et al., 2015). Phages can penetrate rat prostate tissue after their intravenous administration (Letkiewicz et al., 2010b). Moreover, it only took a few minutes for phages to penetrate the circulation after rectal introduction of bacteriophages in an experiment on rabbits and mice. The blood–phage level may be approximately two orders of magnitude higher than that with oral feeding (Letkiewicz et al., 2009). Nguyen et al. estimated that about 31 billion phages penetrate the intestinal epithelial cell of the human body each day (Nguyen et al., 2017). The hemorrhoidal venous plexus, extending along the whole rectum, connects *via* the hemorrhoid genital veins with the prostatic venous plexus, the veins of which are unidirectional (Letkiewicz et al., 2010b). This feature may play a role in genitourinary pathology and may also enable drugs to reach the prostate. All previous statements provide reliable pathways for phages to treat prostatitis. Moreover, rectal administration may also be a more efficient route for phage delivery.

Therapeutic phages are generally regarded as safe (Bruttin and Brussow, 2005; McCallin et al., 2013). Although not enough evidence shows that these phages can cause significant adverse effects or serious harm in mammalian cells, the possibility of bacteriophage-mediated transfer of genes that are involved in bacterial pathogenicity should be a focus of research (Gorski et al., 2009). Generally, only the obligately lytic phages and not lysogenic phages are considered as suitable substrates for the therapeutic phage preparations, because the latter cannot destroy the bacterial cell immediately (Letkiewicz et al., 2010b). As for immunocompromised patients with diseases such as AIDS, the immunological response to phage may be indicative of the potential for an adverse reaction, which could hypothetically worsen a patient’s condition. Currently, there is no consensus on this possibility (Borysowski and Gorski, 2008). A recent study indicated that a serious implication known as “leaky gut” may be linked with the potential of PT to disrupt normal intestinal barrier function in patients with several disorders such as Crohn’s disease, inflammatory bowel disease, and type 1 diabetes (Tetz and Tetz, 2016).

One should be aware that the general use of phages as an alternative to antibiotics will be possible when their efficiency and safety have been verified in large-scale and controlled clinical trials.

### Preventive Action in Plant Extracts

Antibiotics (levofloxacin and other fluoroquinolones) have been recommended to be the preferred treatment for bacterial prostatitis for years. However, the eradication rates of *E. faecalis* and *E. coli* treated with fluoroquinolones vary greatly, ranging between 50%–70% and 70%–90%, respectively (Magri et al., 2011a; Magri et al., 2019). Therefore, much interest has surrounded non-antimicrobial-based approaches. Plant extracts that have long been used for prevention and treatment of urinary tract disorders in traditional medicine are becoming increasingly popular with scholars, especially in this era of antibiotics resistance.

Given that the anti-inflammatory and antibiotic properties of cranberry prevent *E. coli* from adhering to urothelial cells, acidifying the urine, and impair colonization and subsequent infection, the cranberry with oxidation resistance has long been a topic of concern for the prevention of UTIs and bacterial prostatitis (Howell et al., 1998; Habash et al., 1999). Moreover, some authors have reported that the flavonoid, phenolics (as phenolic acids, anthocyanins, and proanthocyanidins), and quercetin are the main compounds of the active components of cranberry. The flavonoid may inhibit free radical and xanthine oxidase activities; thus, it exhibits antibiotic effects and antifungal activity (Shoskes et al., 1999). The phenolics (as phenolic acids, anthocyanins, and proanthocyanidins) are metabolized mainly to hippuric acid with potent anti-adherent and anti-inflammatory effect (Vidlar et al., 2010). Moreover, the quercetin can reduce oxidative stress in prostatitis and has an anti-inflammatory effect (Shoskes et al., 1999). In addition, the combination of various constituents of cranberry may improve their bioactivity due to synergistic effects (Vidlar et al., 2010). An animal model by Kim et al. indicated the preventive potential of the cranberry for CBP by examining their anti-inflammatory activities (Kim et al., 2011). A randomized control trial comparing the dried powdered cranberries (1500 mg per day for 6 months) with no cranberry treatment in 42 participants was the first to evaluate the clinical efficacy of cranberry in the treatment of LUTS, specifically in men with BHP, elevated PSA levels, and prostatitis (Vidlar et al., 2010). Moreover, results suggested that cranberries are highly effective in improving prostate health by relieving PSA elevation in patients with prostatitis and by improving voiding dysfunction, independent of benign prostatic hyperplasia or C-reactive protein level (Vidlar et al., 2010). In addition, the diuretic effects of the cranberry may also have contributed to the reduction in LUTS. Different from currently used antibiotics for prostatitis and LUTS, the main adverse effects of the cranberry are gastrointestinal intolerance, weight gain, and drug–cranberry interactions (Guay, 2009). No date can be quoted to prove that the cranberry can be used to treat UITs or CBP. Therefore, the focus of the cranberry is on its application for prevention.

Kim et al. reported that ginsenoid has preventive effect on patients with CBP (Kim S. H. et al., 2012). A trial comparing probiotics (Bifiprost(R)) + *Serenoa repens* 320 mg versus *S. repens* 320 mg alone in 120 patients with CBP (included for the prevention of CBP) due to *Enterobacteriaceae* showed that the combination of probiotics (Bifiprost(R)) and *S. repens* may prevent the occurrence of episodes of CBP and ameliorate prostatitis-related symptoms after 6 months of therapy (Chiancone et al., 2019).

### Combination Therapies

A multimodal approach to the prolonged antibiotic therapy may be helpful for a higher success rate of BP. Some studies have reported various effective combination therapies for bacterial prostatitis, including plant extracts and antibiotics.

### Plant Extracts Combinate With Antibiotics

The properties of anti-oxidative, anti-carcinogenic, antimicrobial, and anti-inflammatory activities of anthocyanins have a wide range of protective biological effects (Konstantin et al., 2008). The use of anthocyanins extracted from black soybean for CBP was evaluated by Yoon et al. by establishing a rat model in an experiment involving 40 adult male Sprague–Dawley rats (Yoon et al., 2018). Results of the trial indicated that anthocyanins with ciprofloxacin group showed a statistically significant decrease in bacterial growth and improvement in prostatic inflammation compared with the ciprofloxacin group, suggesting that anthocyanins may have anti-inflammatory and antimicrobial effects (Yoon et al., 2018). In addition, a synergistic effect was observed in the anthocyanins plus ciprofloxacin group, which indicated that the combination of anthocyanins and ciprofloxacin may obtain a higher rate of success in treating CBP (Yoon et al., 2018). The report by Sohn et al. indicates that garlic may have anti-inflammatory and antimicrobial effects. The combination of garlic and ciprofloxacin may be effective in treating CBP with a high success rate (Sohn et al., 2009). A prospective randomized study showed that the association of *S. repens*, *Urtica dioica* (ProstaMEV), quercetin (FlogMEV), and curcumin extracts is able to improve the clinical efficacy of prulifloxacin in patients affected by bacterial prostatitis (Cai et al., 2009). Lycopene, an extract of tomatoes, has been reported to have an anti-inflammatory effect by an antioxidative effect and may have an additional (synergistic) effect with ciprofloxacin in the treatment of CBP (Han et al., 2008). Furthermore, the combination of *S. repens*, selenium, lycopene plus bromelain, and methylsulfonylmethane extracts reportedly improves the success rate of levofloxacin in patients with CBP (Cai et al., 2016). Catechin, an extract of green tea, has antimicrobial effect on various bacteria and synergistic effect on antibiotics. In the treatment of CBP in an animal model, the beneficial result of combination treatment of catechin and ciprofloxacin has shown that catechin may be an effective material for treating CBP, and the combination therapy has synergistic effect, thereby suggesting that it may improve clinical efficacy (Lee et al., 2005). However, given that catechin can be easily degenerated during digestion, nanocatechin, which is a catechin coated with hydroxypropyl methyl cellulose by nanotechnology, has attracted researchers’ attention. It reduces degeneration during digestion and enhances absorption of catechin into the body. Yoon has confirmed that nanocatechin has better antimicrobial and anti-inflammatory effects on CBP than catechin via a randomized control trial involving a rat model (Yoon et al., 2011).

### Antibiotics Combinate With Antibiotics

The improvement of eradication rates on bacterial prostatitis through the combination of antibiotics has also been reported by many randomized trials. Magri et al. found that the pathogen eradication under the combination of levofloxacin and azithromycin was 11% increased compared with the cases treated with levofloxacin as a single agent (achieved in 79%) and recommended this as an interesting option in both first-referral and relapsing cases (Magri et al., 2019). Owing to a series of distinct PK and pharmacodynamic properties (including broad antibacterial spectrum, high intracellular accumulation in phagocytes and at sites of infected prostate, biofilm-inhibiting property, immunomodulating effect, and inflammation-resolving activity), macrolide antibiotics are emerging as noteworthy options for enhancing the rates of clinical symptom improvement and pathogen eradication on the treatment of CBP (Perletti et al., 2011). A study of the fluoroquinolone–macrolide combination therapy for CBP has shown that fluoroquinolones combined with macrolides can effectively eliminate pathogenic bacteria and reduce CBP symptoms, such as painful dysuria and sexual dysfunction (Magri et al., 2011b). Khryanin et al. substantiated the superiority of the combination therapy of ornidazole and ofloxacin for CBP, with the background of general decline in sexually transmitted infection incidence (mostly urogenital trichomoniasis) (Khryanin and Reshetnikov, 2016).

### Other Combination Therapy

Administration of immunomodulators must be included in the combination treatment of CBP due to the changes in immunological parameters reflecting the depression of the immune system (Kamalov et al., 2010). In a cohort of recurrent chronic prostatitis patients (RCBP) treated with immunomodulator Panavir, Novikov and his coworkers reported that the addition of Panavir to standard treatment of RCBP patients significantly improved treatment results. Panavir is recommended as an adjuvant in combined RCBP treatment (Novikov et al., 2010).

Androgen directly controls the growth and development of the prostate gland. Thus, prostatitis may be also directly influenced by hormone milieu, similar to the progress of BPH and PCA. Therefore, the effects of androgen deprivation on the treatment of CBP were investigated in rats. Result of the trial reported by Seo et al. showed that the finasteride and levofloxacin groups showed significant decreases in bacterial growth and great improvements in prostatic inflammation compared with the control group. This finding suggested that androgen deprivation is an effective modality in treatment of CBP, and the combination of finasteride and levofloxacin may be one of the effective treatment modalities (Seo et al., 2003). In addition, Lee used an animal model and suggested that finasteride may have a preventive effect on development of CBP, although there is no consensus yet on the mechanism of this effect (Lee et al., 2011).

Apart from the aforementioned combination therapy, Aliaev et al. reported the significant rising of efficacy in treating CBP with the combination of antibacterial and vardenafil (Aliaev et al., 2008). Moreover, the interaction between levofloxacin and diclofenac sodium has been proved by Fayyaz et al. (Fayyaz et al., 2015). Studies have shown that selenium decreased bacterial infection significantly, and the therapy administered by both selenium and an antibiotic was more effective than the therapy that used only one of the agents to hinder bacterial infection on prostate tissue (Kim H. W. et al., 2012).

## Other Treatment Regimens

The confirmation that a low-intensity electromagnetic radiation in the microwave range with a frequency of 1 GHz has anti-inflammatory and trophic effects in a number of inflammatory diseases provides a basis for resonance-microwave therapy for CBP. Furthermore, beneficial effect of the low-intensity resonance-microwave therapy is seen on the levels of blood proinflammatory cytokines in patients with CBP. The present study has demonstrated that resonance-microwave therapy increased clinical efficacy of patients with CBP compared with baseline drug therapy. Therefore, low-intensity electromagnetic radiation with a frequency of 1 GHz is confirmed to be feasible for the treatment of CBP (Kiyatkin et al., 2015).

Studies have suggested that microbubble-mediated ultrasound irradiation significantly increased the permeability of prostate tissue and the effective concentrations of drugs in local tissue (Li et al., 2012; Liu et al., 2013). In addition, Yi et al. have demonstrated that the effect of inhibiting inflammation and reducing TNF-alpha and IL-1beta expressions on the prostate tissues can be observed by microbubble-mediated ultrasound-induced accumulation of bone marrow mesenchymal stem cell (BMMSCs), thereby suggesting that this method may be effective for CBP (Yi et al., 2016). Schoeb et al. performed a systematic literature search and suggested that surgical therapy of CBP might be a viable option. However, further evidence and randomized controlled trials are needed to provide a basis for clinical decisions (Schoeb et al., 2017). Transurethral using the double-balloon and triple-channel catheter has a better clinical efficacy and has the obvious advantages of being safe, effective, easy, and repeatable when combined with other hypurgia compared with traditional intravenous treatment for CBP (Huang et al., 2003). Some case reports showed that injecting antibiotics directly into the prostate gland was obviously effective, but further research evidences are needed to accept this approach.

## Conclusions

Although global medical technology is rapidly developing in the new century, there is still a long way to go in the prevention and treatment of bacterial prostatitis especially chronic bacterial prostatitis. There is a relationship between ABP and CBP. Approximately 10% of ABP will progress to CBP. Treatment of CBP is complicated due to the presence of multidrug-resistant bacteria, EBSL-producing *E. coli*, biofilms-producing bacteria, and the shift in bacterial etiology. Therefore, appropriate and personalized treatment schedule after bacterial culture and drug susceptibility test is recommended instead of empirical medication. New therapeutic strategies including fosfomycin, therapeutic strategy and combination therapies have led to a dramatic increase in bacterial-eradication rates and a qualitative improvement in patients’ quality of life. Although the management of bacterial prostatitis is difficult at present, this article aims to give some new treatment ideas to urologist to replace traditional treatment. Further research into risk factors, pathogenesis, diagnosis, prevention, and treatment should be conducted in the future to address the current challenges. Bacterial prostatitis will be no longer a common disease that confuses doctors and patients.

## Author Contributions

SX searched the literature and conceived and wrote the review. LX, DW, ZZ, LY, TY, CL, WG and FB critically appraised the literature and made a intellectual contribution to the work. All authors approved the final version of the manuscript for publication.

## Funding

The present study was supported by the National Natural Science Foundation of China (grant no. 81560419 and no. 81960512) and the Natural Science Foundation of Jiangxi (grant no. 20151BAB205047).

## Conflict of Interest

The authors declare that the research was conducted in the absence of any commercial or financial relationships that could be construed as a potential conflict of interest.

## References

[B1] Ackerman A. L. Parameshwar P. S. Anger J. T. (2018). Diagnosis and treatment of patients with prostatic abscess in the post-antibiotic era. Int. J. Urol. 25, 103–110. 10.1111/iju.13451 28944509

[B2] Aliaev I. Vinarov A. Z. Akhvlediani N. D. (2008). Wardenafil in combined treatment of patients with chronic bacterial prostatitis. Urologiia. 6, 52–55. 19256057

[B3] Almeida F. Santos S. A. Silva P. A. Sarmento A. (2019). Chronic prostatitis caused by extended-spectrum beta-lactamase-producing Escherichia coli managed using oral fosfomycin-A case report. IDCases 15, e493. 10.1016/j.idcr.2019.e00493 PMC6360601 30766796

[B4] Andrea H. Michael B. Valerie S. Anneta R. (2003). Role of capsular colanic acid in adhesion of uropathogenic Escherichia coli. Appl. Environ. Microbiol. 69, 4474. 10.1128/AEM.69.8.4474-4481.2003 12902231 PMC169069

[B5] Aravantinos E. Kalogeras N. Zygoulakis N. Kakkas G. Anagnostou T. Meleskos M. (2008). Ultrasound-guided transrectal placement of a drainage tube as therapeutic management of patients with prostatic abscess. J. Endourol. 22, 1751–1754. 10.1089/end.2008.0265 18673079

[B6] Bartoletti R. Cai T. Nesi G. Albanese S. Meacci F. Mazzoli S. (2014). The impact of biofilm-producing bacteria on chronic bacterial prostatitis treatment: Results from a longitudinal cohort study. World J. Urol. 32, 737–742. 10.1007/s00345-013-1145-9 23918259

[B7] Benway B. M. Moon T. D. (2008). Bacterial prostatitis. Urol. Clin. North Am. 35, 23–32. 10.1016/j.ucl.2007.09.008 18061021

[B8] Biswas B. Adhya S. Washart P. Paul B. Trostel A. N. Powell B. (2002). Bacteriophage therapy rescues mice bacteremic from a clinical isolate of vancomycin-resistant Enterococcus faecium. Infect. Immun. 70, 204–210. 10.1128/iai.70.1.204-210.2002 11748184 PMC127648

[B9] Borysowski J. Gorski A. (2008). Is phage therapy acceptable in the immunocompromised host? Int. J. Infect. Dis. 12, 466–471. 10.1016/j.ijid.2008.01.006 18400541

[B10] Bourdin G. Navarro A. Sarker S. A. Pittet A. C. Qadri F. Sultana S. (2014). Coverage of diarrhoea-associated Escherichia coli isolates from different origins with two types of phage cocktails. Microb. Biotechnol. 7, 165–176. 10.1111/1751-7915.12113 24528873 PMC3937720

[B11] Brede C. M. Shoskes D. A. (2011). The etiology and management of acute prostatitis. Nat. Rev. Urol. 8, 207–212. 10.1038/nrurol.2011.22 21403661

[B12] Bruttin A. Brussow H. (2005). Human volunteers receiving Escherichia coli phage T4 orally: A safety test of phage therapy. Antimicrob. Agents Chemother. 49, 2874–2878. 10.1128/AAC.49.7.2874-2878.2005 15980363 PMC1168693

[B13] Bundrick W. Heron S. P. Ray P. Schiff W. M. Tennenberg A. M. Wiesinger B. A. (2003). Levofloxacin versus ciprofloxacin in the treatment of chronic bacterial prostatitis: A randomized double-blind multicenter study. Urology 62, 537–541. 10.1016/s0090-4295(03)00565-x 12946763

[B14] Cai T. Mazzoli S. Bechi A. Addonisio P. Mondaini N. Pagliai R. C. (2009). Serenoa repens associated with Urtica dioica (ProstaMEV) and curcumin and quercitin (FlogMEV) extracts are able to improve the efficacy of prulifloxacin in bacterial prostatitis patients: Results from a prospective randomised study. Int. J. Antimicrob. Agents 33, 549–553. 10.1016/j.ijantimicag.2008.11.012 19181486

[B15] Cai T. Mazzoli S. Meacci F. Boddi V. Mondaini N. Malossini G. (2011). Epidemiological features and resistance pattern in uropathogens isolated from chronic bacterial prostatitis. J. Microbiol. 49, 448–454. 10.1007/s12275-011-0391-z 21717331

[B16] Cai T. Pisano F. Magri V. Verze P. Mondaini N. D'Elia C. (2014). Chlamydia trachomatis infection is related to premature ejaculation in chronic prostatitis patients: Results from a cross-sectional study. J. Sex Med. 11, 3085–3092. 10.1111/jsm.12699 25256084

[B17] Cai T. Tiscione D. Gallelli L. Verze P. Palmieri A. Mirone V. (2016). Serenoa repens associated with selenium and lycopene extract and bromelain and methylsulfonylmethane extract are able to improve the efficacy of levofloxacin in chronic bacterial prostatitis patients. Arch. Ital Urol. Androl. 88, 177–182. 10.4081/aiua.2016.3.177 27711089

[B18] Cai T. Tessarolo F. Caola I. Piccoli F. Nollo G. Caciagli P. (2018). Prostate calcifications: A case series supporting the microbial biofilm theory. Invest. Clin. Urol. 59, 187–193. 10.4111/icu.2018.59.3.187 PMC5934281 29744476

[B19] Cek M. Lenk S. Naber K. G. Bishop M. C. Johansen T. E. (2005). EAU guidelines for the management of genitourinary tuberculosis. Eur. Urol. 48, 353–362. 10.1016/j.eururo.2005.03.008 15982799

[B20] Centers for Disease Control and Prevention (2013). Antibiotic resistance threats in the United States, 2013. http://www.cdc.gov/drugresistance/threat-report-2013/ [Accessed April 23, 2013].

[B21] Chiancone F. Carrino M. Meccariello C. Pucci L. Fedelini M. Fedelini P. (2019). The use of a combination of vaccinium macracarpon, lycium barbarum l. And probiotics (Bifiprost®) for the prevention of chronic bacterial prostatitis: A Double-Blind randomized study. Urol. Int. 4, 1–4. 10.1159/000502765 31527377

[B22] Chibani-Chennoufi S. Sidoti J. Bruttin A. Kutter E. Sarker S. Brussow H. (2004). In vitro and in vivo bacteriolytic activities of Escherichia coli phages: Implications for phage therapy. Antimicrob. Agents Chemother. 48, 2558–2569. 10.1128/AAC.48.7.2558-2569.2004 15215109 PMC434175

[B23] Chou Y. H. Tiu C. M. Liu J. Y. Chen J. D. Chiou H. J. Chiou S. Y. (2004). Prostatic abscess: Transrectal color Doppler ultrasonic diagnosis and minimally invasive therapeutic management. Ultrasound Med. Biol. 30, 719–724. 10.1016/j.ultrasmedbio.2004.03.014 15219951

[B24] Coker T. J. Dierfeldt D. M. (2016). Acute bacterial prostatitis: Diagnosis and management. Am. Fam. Physician 93, 114–120. 26926407

[B25] Collins M. M. Stafford R. S. O’Leary M. P. Barry M. J. (1998). How common is prostatitis? A national survey of physician visits. J. Urol. 159, 1224–1228. 10.1016/S0022-5347(01)63564-X 9507840

[B26] Comeau A. M. Tetart F. Trojet S. N. Prere M. F. Krisch H. M. (2007). Phage-Antibiotic Synergy (PAS): Beta-lactam and quinolone antibiotics stimulate virulent phage growth. PloS One 2, e799. 10.1371/journal.pone.0000799 17726529 PMC1949050

[B27] Corvec S. Furustrand T. U. Betrisey B. Borens O. Trampuz A. (2013). Activities of fosfomycin, tigecycline, colistin, and gentamicin against extended-spectrum-beta-lactamase-producing Escherichia coli in a foreign-body infection model. Antimicrob. Agents Chemother. 57, 1421–1427. 10.1128/AAC.01718-12 23295934 PMC3591882

[B28] Costelloe C. Metcalfe C. Lovering A. Mant D. Hay A. D. (2010). Effect of antibiotic prescribing in primary care on antimicrobial resistance in individual patients: Systematic review and meta-analysis. Bmj 340, c2096. 10.1136/bmj.c2096 20483949

[B29] Costerton J. W. Lewandowski Z. Caldwell D. E. Korber D. R. Lappin-Scott H. M. (1995). Microbial biofilms. Annu. Rev. Microbiol. 49, 711–745. 10.1146/annurev.mi.49.100195.003431 8561477

[B30] Cunha B. A. Gran A. Raza M. (2015). Persistent extended-spectrum beta-lactamase-positive Escherichia coli chronic prostatitis successfully treated with a combination of fosfomycin and doxycycline. Int. J. Antimicrob. Agents 45, 427–429. 10.1016/j.ijantimicag.2014.12.019 25662814

[B31] de la Rosette J. J. Hubregtse M. R. Meuleman E. J. Stolk-Engelaar M. V. Debruyne F. M. (1993). Diagnosis and treatment of 409 patients with prostatitis syndromes. Urology 41, 301–307. 10.1016/0090-4295(93)90584-w 8470312

[B32] Denisuik A. J. Lagace-Wiens P. R. Pitout J. D. Mulvey M. R. Simner P. J. Tailor F. (2013). Molecular epidemiology of extended-spectrum beta-lactamase-, AmpC beta-lactamase- and carbapenemase-producing Escherichia coli and Klebsiella pneumoniae isolated from Canadian hospitals over a 5 year period: CANWARD 2007-11. J. Antimicrob. Chemother. 68 Suppl 1, i57–i65. 10.1093/jac/dkt027 23587779

[B33] Doi Y. Park Y. S. Rivera J. I. Adams-Haduch J. M. Hingwe A. Sordillo E. M. (2013). Community-associated extended-spectrum beta-lactamase-producing Escherichia coli infection in the United States. Clin. Infect. Dis. 56, 641–648. 10.1093/cid/cis942 23150211 PMC3563390

[B34] Donlan R. M. (2009). Preventing biofilms of clinically relevant organisms using bacteriophage. Trends Microbiol. 17, 66–72. 10.1016/j.tim.2008.11.002 19162482

[B35] Doolittle M. M. Cooney J. J. Caldwell D. E. (1995). Lytic infection of Escherichia coli biofilms by bacteriophage T4. Can. J. Microbiol. 41, 12–18. 10.1139/m95-002 7728652

[B36] Etienne M. Chavanet P. Sibert L. Michel F. Levesque H. Lorcerie B. (2008). Acute bacterial prostatitis: Heterogeneity in diagnostic criteria and management. Retrospective multicentric analysis of 371 patients diagnosed with acute prostatitis. BMC Infect. Dis. 8, 12. 10.1186/1471-2334-8-12 18234108 PMC2254416

[B37] Fair W. R. Parrish R. F. (1981). Antibacterial substances in prostatic fluid. Prog. Clin. Biol. Res. 75A, 247–264. 7041133

[B38] Falagas M. E. Rafailidis P. I. (2015). Editorial commentary: Fosfomycin: The current status of the drug. Clin. Infect. Dis. 61, 1144–1146. 10.1093/cid/civ443 26063717

[B39] Fan L. Shang X. Zhu J. Ma B. Zhang Q. (2018). Pharmacodynamic and pharmacokinetic studies and prostatic tissue distribution of fosfomycin tromethamine in bacterial prostatitis or normal rats. Andrologia 50, e13021. 10.1111/and.13021 29718594

[B40] Fayyaz M. Yousuf R. I. Shoaib M. H. Ali T. Nasiri I. Ashraf N. (2015). Quality evaluation and in vitro interaction studies between levofloxacin 250mg and diclofenac sodium 50mg tablets. Pak J. Pharm. Sci. 28, 119–128. 10.3906/yer-1412-34 25553690

[B41] Garcia-Castillo M. Morosini M. I. Galvez M. Baquero F. Del C. R. Meseguer M. A. (2008). Differences in biofilm development and antibiotic susceptibility among clinical Ureaplasma urealyticum and Ureaplasma parvum isolates. J. Antimicrob. Chemother. 62, 1027–1030. 10.1093/jac/dkn337 18753188

[B42] Gardiner B. J. Mahony A. A. Ellis A. G. Lawrentschuk N. Bolton D. M. Zeglinski P. T. (2014). Is fosfomycin a potential treatment alternative for multidrug-resistant gram-negative prostatitis? Clin. Infect. Dis. 58, e101–e105. 10.1093/cid/cit704 24170195

[B43] Gill B. C. Shoskes D. A. (2016). Bacterial prostatitis. Curr. Opin. Infect. Dis. 29, 86–91. 10.1097/QCO.0000000000000222 26555038

[B44] Gorski A. Miedzybrodzki R. Borysowski J. Weber-Dabrowska B. Lobocka M. Fortuna W. (2009). Bacteriophage therapy for the treatment of infections. Curr. Opin. Invest. Drugs 10, 766–774. 10.1117/12.895292 19649921

[B45] Gorski A. Dabrowska K. Hodyra-Stefaniak K. Borysowski J. Miedzybrodzki R. Weber–Dabrowska B. (2015). Phages targeting infected tissues: Novel approach to phage therapy. Future Microbiol. 10, 199–204. 10.2217/fmb.14.126 25689532

[B46] Goto T. Makinose S. Ohi Y. Yamauchi D. Kayajima T. Nagayama K. (1998). Diffusion of piperacillin, cefotiam, minocycline, amikacin and ofloxacin into the prostate. Int. J. Urol. 5, 243–246. 10.1111/j.1442-2042.1998.tb00597.x 9624555

[B47] Goyal N. K. Goel A. Sankhwar S. Dalela D. (2013). Transurethral resection of prostate abscess: Is it different from conventional transurethral resection for benign prostatic hyperplasia? ISRN Urol. 2013, 109505. 10.1155/2013/109505 23840969 PMC3693178

[B48] Guay D. R. (2009). Cranberry and urinary tract infections. Drugs 69, 775–807. 10.2165/00003495-200969070-00002 19441868

[B49] Habash M. B. Van der Mei H. C. Busscher H. J. Reid G. (1999). The effect of water, ascorbic acid, and cranberry derived supplementation on human urine and uropathogen adhesion to silicone rubber. Can. J. Microbiol. 45, 691–694. 10.1139/w99-065 10528401

[B50] Hallgren A. Abednazari H. Ekdahl C. Hanberger H. Nilsson M. Samuelsson A. (2001). Antimicrobial susceptibility patterns of enterococci in intensive care units in Sweden evaluated by different MIC breakpoint systems. J. Antimicrob. Chemother. 48, 53–62. 10.1093/jac/48.1.53 11418512

[B51] Han C. H. Yang C. H. Sohn D. W. Kim S. W. Kang S. H. Cho Y. H. (2008). Synergistic effect between lycopene and ciprofloxacin on a chronic bacterial prostatitis rat model. Int. J. Antimicrob. Agents 31 Suppl 1, S102–S107. 10.1016/j.ijantimicag.2007.07.016 17920247

[B52] Hanlon G. W. (2007). Bacteriophages: An appraisal of their role in the treatment of bacterial infections. Int. J. Antimicrob. Agents 30, 118–128. 10.1016/j.ijantimicag.2007.04.006 17566713

[B53] Hatfull G. F. (2008). Bacteriophage genomics. Curr. Opin. Microbiol. 11, 447–453. 10.1016/j.mib.2008.09.004 18824125 PMC2706577

[B54] Heras-Canas V. Gutierrez-Soto B. Serrano-Garcia M. L. Vazquez-Alonso F. Navarro-Mari J. M. Gutierrez–Fernandez J. (2016). Chronic bacterial prostatitis. Clinical and microbiological study of 332 cases. Med. Clin. (Barc) 147, 144–147. 10.1016/j.medcli.2016.05.018 27377215

[B55] Hoban D. J. Lascols C. Nicolle L. E. Badal R. Bouchillon S. Hackel M. (2012). Antimicrobial susceptibility of Enterobacteriaceae, including molecular characterization of extended-spectrum beta-lactamase-producing species, in urinary tract isolates from hospitalized patients in North America and Europe: Results from the SMART study 2009-2010. Diagn. Microbiol. Infect. Dis. 74, 62–67. 10.1016/j.diagmicrobio.2012.05.024 22763019

[B56] Howell A. B. Vorsa N. Der Marderosian A. Foo L. Y. (1998). Inhibition of the adherence of P-fimbriated Escherichia coli to uroepithelial-cell surfaces by proanthocyanidin extracts from cranberries. N. Engl. J. Med. 339, 1085–1086. 10.1056/NEJM199810083391516 9767006

[B57] Huang W. D. Huang W. J. Liu P. Ren W. Xu B. Wang X. J. (2003). Treatment of chronic bacterial prostatitis by perfusion with double-balloon and triple-channel catheter: A control study. Zhonghua Nan Ke Xue 9, 580–583. 14689888

[B58] Hughes K. A. Sutherland I. W. Jones M. V. (1998). Biofilm susceptibility to bacteriophage attack: The role of phage-borne polysaccharide depolymerase. Microbiology 144 ( Pt 11), 3039–3047. 10.1099/00221287-144-11-3039 9846739

[B59] Hurtado F. K. Laureano J. V. de A L. G. Derendorf H. Dalla C. T. (2014). Enhanced penetration of moxifloxacin into rat prostate tissue evidenced by microdialysis. Int. J. Antimicrob. Agents 44, 327–333. 10.1016/j.ijantimicag.2014.06.011 25218157

[B60] Hyman P. Abedon S. T. (2010). Bacteriophage host range and bacterial resistance. Adv. Appl. Microbiol. 70, 217–248. 10.1016/S0065-2164(10)70007-1 20359459

[B61] Ivanov I. B. Gritsenko V. A. Kuzmin M. D. (2008). Distribution of secretory inhibitor of platelet microbicidal protein among urethral isolates with its correlation with prostatitis. Asian J. Androl. 10, 189–192. 10.1111/j.1745-7262.2008.00344.x 18097515

[B62] Kamalov A. A. Efremov E. A. Dorofeev S. D. Tret’Iakov A. A. Okhobotov D. A. Mel'nik IaI (2010). Clinical immunological rationale of interferon therapy in chronic bacterial prostatitis. Urologiia 1, 34–38. 20886729

[B63] Kanamaru S. Kurazono H. Terai A. Monden K. Kumon H. Mizunoe Y. (2006). Increased biofilm formation in Escherichia coli isolated from acute prostatitis. Int. J. Antimicrob. Agents 28 Suppl 1, S21–S25. 10.1016/j.ijantimicag.2006.05.006 16828264

[B64] Kanjanawongdeengam P. Viseshsindh W. Santanirand P. Prathombutr P. Nilkulwattana S. (2009). Reduction in bacteremia rates after rectum sterilization before transrectal, ultrasound-guided prostate biopsy: A randomized controlled trial. J. Med. Assoc. Thai 92, 1621–1626. 20043564

[B65] Karaiskos I. Galani L. Sakka V. Gkoufa A. Sopilidis O. Chalikopoulos D. (2019). Oral fosfomycin for the treatment of chronic bacterial prostatitis. J. Antimicrob. Chemother. 74, 1430–1437. 10.1093/jac/dkz015 30796442 PMC6477975

[B66] Karlowsky J. A. Lagace-Wiens P. R. Simner P. J. DeCorby M. R. Adam H. J. Walkty A. (2011). Antimicrobial resistance in urinary tract pathogens in Canada from 2007 to 2009: CANWARD surveillance study. Antimicrob. Agents Chemother. 55, 3169–3175. 10.1128/AAC.00066-11 21537027 PMC3122429

[B67] Karlowsky J. A. Denisuik A. J. Lagace-Wiens P. R. Adam H. J. Baxter M. R. Hoban D. J. (2014). In Vitro activity of fosfomycin against Escherichia coli isolated from patients with urinary tract infections in Canada as part of the CANWARD surveillance study. Antimicrob. Agents Chemother. 58, 1252–1256. 10.1128/AAC.02399-13 24323476 PMC3910835

[B68] Khan F. U. Ihsan A. U. Khan H. U. Jana R. Wazir J. Khongorzul P. (2017). Comprehensive overview of prostatitis. BioMed. Pharmacother. 94, 1064–1076. 10.1016/j.biopha.2017.08.016 28813783

[B69] Khryanin A. A. Reshetnikov O. V. (2016). Combination therapy of chronic bacterial prostatitis. Urologiia 3(Supply 3), 91–96. 28247621

[B70] Kim S. H. Ha U. S. Lee H. R. Sohn D. W. Lee S. J. Kim H. W. (2011). Do Escherichia coli extract and cranberry exert preventive effects on chronic bacterial prostatitis? Pilot study using an animal model. J. Infect. Chemother. 17, 322–326. 10.1007/s10156-010-0170-5 21042827

[B71] Kim H. W. Ha U. S. Woo J. C. Kim S. J. Yoon B. I. Lee S. J. (2012). Preventive effect of selenium on chronic bacterial prostatitis. J. Infect. Chemother. 18, 30–34. 10.1007/s10156-011-0276-4 21814801

[B72] Kim S. H. Ha U. S. Sohn D. W. Lee S. J. Kim H. W. Han C. H. (2012). Preventive effect of ginsenoid on chronic bacterial prostatitis. J. Infect. Chemother. 18, 709–714. 10.1007/s10156-012-0406-7 22450878

[B73] Kim S. H. Ha U. S. Yoon B. I. Kim S. W. Sohn D. W. Kim H. W. (2014). Microbiological and clinical characteristics in acute bacterial prostatitis according to lower urinary tract manipulation procedure. J. Infect. Chemother. 20, 38–42. 10.1016/j.jiac.2013.11.004 24462423

[B74] Kiyatkin V. A. Konchugova T. V. Yakovlev M. Y. Bobkov A. D. (2015). The application of the combined resonance-wave therapy for the treatment of the patients presenting with chronic bacterial prostatitis. Vopr Kurortol. Fizioter Lech Fiz Kult 92, 40–44. 10.17116/kurort2015540-44 26852501

[B75] Konstantin T. Hyeong Bin P. Young Min K. Jong Il C. Sung Chul S. Won Suk L. (2008). Anthocyanins from black soybean seed coats inhibit UVB-induced inflammatory cylooxygenase-2 gene expression and PGE2 production through regulation of the nuclear factor-kappaB and phosphatidylinositol 3-kinase/Akt pathway. J. Agric. Food Chem. 56, 8969–8974. 10.1021/jf801345c 18778065

[B76] Krieger J. N. Egan K. J. (1991). Comprehensive evaluation and treatment of 75 men referred to chronic prostatitis clinic. Urology 38(1), 9–11. 10.1016/0090-4295(91)80004-q 1866851

[B77] Krieger J. N. Nyberg L. J. Nickel J. C. (1999). NIH consensus definition and classification of prostatitis. Jama 282, 236–237. 10.1001/jama.282.3.236 10422990

[B78] Kurzer E. Kaplan S. (2002). Cost effectiveness model comparing trimethoprim sulfamethoxazole and ciprofloxacin for the treatment of chronic bacterial prostatitis. Eur. Urol. 42, 163–166. 10.1016/S0302-2838(02)00270-1 12160588

[B79] Labrie S. J. Samson J. E. Moineau S. (2010). Bacteriophage resistance mechanisms. Nat. Rev. Microbiol. 8, 317–327. 10.1038/nrmicro2315 20348932

[B80] Laxminarayan R. Amabile-Cuevas C. F. Cars O. Evans T. Heymann D. L. Hoffman S. (2016). UN High-Level Meeting on antimicrobials–what do we need? Lancet 388, 218–220. 10.1016/S0140-6736(16)31079-0 27479554

[B81] Lee Y. S. Han C. H. Kang S. H. Lee S. J. Kim S. W. Shin O. R. (2005). Synergistic effect between catechin and ciprofloxacin on chronic bacterial prostatitis rat model. Int. J. Urol. 12, 383–389. 10.1111/j.1442-2042.2005.01052.x 15948727

[B82] Lee C. B. Ha U. S. Yim S. H. Lee H. R. Sohn D. W. Han C. H. (2011). Does finasteride have a preventive effect on chronic bacterial prostatitis? Pilot study using an animal model. Urol. Int. 86, 204–209. 10.1159/000320109 21273757

[B83] Letkiewicz S. Miedzybrodzki R. Fortuna W. Weber-Dabrowska B. Gorski A. (2009). Eradication of Enterococcus faecalis by phage therapy in chronic bacterial prostatitis–case report. Folia Microbiol. (Praha) 54, 457–461. 10.1007/s12223-009-0064-z 19937220

[B84] Letkiewicz S. Miedzvbrodzki R. Klak M. Dabrowska B. W. Gorski A. (2010a). Pathogen eradication by phage therapy in patients with chronic bacterial prostatitis. Eur. Urol. Suppl. 9, 140. 10.1016/S1569-9056(10)60372-7

[B85] Letkiewicz S. Miedzybrodzki R. Klak M. Jonczyk E. Weber-Dabrowska B. Gorski A. (2010b). The perspectives of the application of phage therapy in chronic bacterial prostatitis. FEMS Immunol. Med. Microbiol. 60, 99–112. 10.1111/j.1574-695X.2010.00723.x 20698884

[B86] Levin B. R. Bull J. J. (2004). Population and evolutionary dynamics of phage therapy. Nat. Rev. Microbiol. 2, 166–173. 10.1038/nrmicro822 15040264

[B87] Li T. Liu G. C. Li J. Wang X. Liu Q. Q. Liu Z. (2012). Mechanisms of prostate permeability triggered by microbubble-mediated acoustic cavitation. Cell Biochem. Biophysics 64, 147–153. 10.1007/s12013-012-9383-9 22722876

[B88] Lin D. M. Koskella B. Lin H. C. (2017). Phage therapy: An alternative to antibiotics in the age of multi-drug resistance. World J. Gastrointest Pharmacol. Ther. 8, 162–173. 10.4292/wjgpt.v8.i3.162 28828194 PMC5547374

[B89] Lipsky B. A. Byren I. Hoey C. T. (2010). Treatment of bacterial prostatitis. Clin. Infect. Dis. 50, 1641–1652. 10.1086/652861 20459324

[B90] Liu Y. L. Yi S. H. Zhang J. L. Fang Z. Q. Zhou F. Jia W. S. (2013). Effect of microbubble-enhanced ultrasound on prostate permeability: A potential therapeutic method for prostate disease. Urology 81, 921. 10.1016/j.urology.2012.12.022 23414693

[B91] Lou J. G. Dong J. Zheng Y. C. Zhang S. M. Xiao W. Q. Zhou J. F. (2006). Increased oxidative stress and damage in patients with chronic bacterial prostatitis. BioMed. Environ. Sci. 19, 481–486. 10.1111/j.1467-842X.2006.tb00794.x 17319275

[B92] Lu T. K. Collins J. J. (2007). Dispersing biofilms with engineered enzymatic bacteriophage. Proc. Natl. Acad. Sci. U. S. A 104, 11197–11202. 10.1073/pnas.0704624104 17592147 PMC1899193

[B93] Ludwig M. (2008). Diagnosis and therapy of acute prostatitis, epididymitis and orchitis. Andrologia 40, 76–80. 10.1111/j.1439-0272.2007.00823.x 18336454

[B94] Magiorakos A. P. Srinivasan A. Carey R. B. Carmeli Y. Falagas M. E. Giske C. G. (2012). Multidrug-resistant, extensively drug-resistant and pandrug-resistant bacteria: An international expert proposal for interim standard definitions for acquired resistance. Clin. Microbiol. Infect. 18, 268–281. 10.1111/j.1469-0691.2011.03570.x 21793988

[B95] Magri V. Wagenlehner F. M. Montanari E. Marras E. Orlandi V. Restelli A. (2009). Semen analysis in chronic bacterial prostatitis: Diagnostic and therapeutic implications. Asian J. Androl. 11, 461–477. 10.1038/aja.2009.5 19377490 PMC3735310

[B96] Magri V. Marras E. Perletti G. (2011a). Chronic bacterial prostatitis: Enterococcal disease? Clin. Infect. Dis. 53, 1307–1308. 10.1093/cid/cir709 22080126

[B97] Magri V. Montanari E. Skerk V. Markotic A. Marras E. Restelli A. (2011b). Fluoroquinolone-macrolide combination therapy for chronic bacterial prostatitis: Retrospective analysis of pathogen eradication rates, inflammatory findings and sexual dysfunction. Asian J. Androl. 13, 819–827. 10.1038/aja.2011.36 21765442 PMC3739561

[B98] Magri V. Perletti G. Cai T. Stamatiou K. Trinchieri A. Montanari E. (2019). Levofloxacin for NIH category II chronic bacterial prostatitis: A Real-Life study. Chemotherapy 64, 8–16. 10.1159/000499034 31112957

[B99] Mazzoli S. (2010). Biofilms in chronic bacterial prostatitis (NIH-II) and in prostatic calcifications. FEMS Immunol. Med. Microbiol. 59, 337–344. 10.1111/j.1574-695X.2010.00659.x 20298500

[B100] McCallin S. Alam S. S. Barretto C. Sultana S. Berger B. Huq S. (2013). Safety analysis of a Russian phage cocktail: From metagenomic analysis to oral application in healthy human subjects. Virology 443, 187–196. 10.1016/j.virol.2013.05.022 23755967

[B101] Michalopoulos A. S. Livaditis I. G. Gougoutas V. (2011). The revival of fosfomycin. Int. J. Infect. Dis. 15, e732–e739. 10.1016/j.ijid.2011.07.007 21945848

[B102] Miedzybrodzki R. Switala-Jelen K. Fortuna W. Weber-Dabrowska B. Przerwa A. Luisak–Szelachowska M. (2008). Bacteriophage preparation inhibition of reactive oxygen species generation by endotoxin-stimulated polymorphonuclear leukocytes. Virus Res. 131, 233–242. 10.1016/j.virusres.2007.09.013 17996972

[B103] Miedzybrodzki R. Fortuna W. Weber-Dabrowska B. Gorski A. (2009). A retrospective analysis of changes in inflammatory markers in patients treated with bacterial viruses. Clin. Exp. Med. 9, 303–312. 10.1007/s10238-009-0044-2 19350363

[B104] Millan-Rodriguez F. Palou J. Bujons-Tur A. Musquera-Felip M. Sevilla-Cecilia C. Serrallach–Orejas M. (2006). Acute bacterial prostatitis: Two different sub-categories according to a previous manipulation of the lower urinary tract. World J. Urol. 24, 45–50. 10.1007/s00345-005-0040-4 16437219

[B105] Miura T. Tanaka K. Shigemura K. Nakano Y. Takenaka A. Fujisawa M. (2008). Levofloxacin resistant Escherichia coli sepsis following an ultrasound-guided transrectal prostate biopsy: Report of four cases and review of the literature. Int. J. Urol. 15, 457–459. 10.1111/j.1442-2042.2007.01975.x 18452466

[B106] Munoz-Price L. S. Poirel L. Bonomo R. A. Schwaber M. J. Daikos G. L. Cormican M. (2013). Clinical epidemiology of the global expansion of Klebsiella pneumoniae carbapenemases. Lancet Infect. Dis. 13, 785–796. 10.1016/S1473-3099(13)70190-7 23969216 PMC4673667

[B107] Naber K. G. Sorgel F. (2003). Antibiotic therapy–rationale and evidence for optimal drug concentrations in prostatic and seminal fluid and in prostatic tissue. Andrologia 35, 331–335. 10.1111/j.1439-0272.2003.tb00868.x 14535866

[B108] Naber K. G. Kinzig M. Sorgel F. Weigel D. (1993). Penetration of ofloxacin into prostatic fluid, ejaculate and seminal fluid. Infection 21, 98–100. 10.1007/bf01710740 8491527

[B109] Naber C. K. Steghafner M. Kinzig-Schippers M. Sauber C. S Rgel F. Stahlberg H. J. (2001). Concentrations of gatifloxacin in plasma and urine and penetration into prostatic and seminal fluid, ejaculate, and sperm cells after single oral administrations of 400 milligrams to volunteers. Antimicrobial Agents Chemother. 45, 293–297. 10.1128/AAC.45.1.293-297.2001 PMC90275 11120980

[B110] Naber K. G. Roscher K. Botto H. Schaefer V. (2008). Oral levofloxacin 500 mg once daily in the treatment of chronic bacterial prostatitis. Int. J. Antimicrob. Agents 32, 145–153. 10.1016/j.ijantimicag.2008.03.014 18571904

[B111] Nagy V. Kubej D. (2012). Acute bacterial prostatitis in humans: Current microbiological spectrum, sensitivity to antibiotics and clinical findings. Urol. Int. 89, 445–450. 10.1159/000342653 23095643

[B112] Neimark A. I. Iurova V. A. Neimark B. A. Aliev R. T. (2010). Characteristic of gram-positive microorganisms isolated during chronic bacterial prostatitis. Zh Mikrobiol. Epidemiol. Immunobiol. 5, 73–77. 21061581

[B113] Nguyen S. Baker K. Padman B. S. Patwa R. Dunstan R. A. Weston T. A. (2017). Bacteriophage transcytosis provides a mechanism to cross epithelial cell layers. MBio 8, e01874–17. 10.1128/mBio 29162715 PMC5698557

[B114] Nickel J. C. Touma N. (2012). Alpha-Blockers for the treatment of chronic Prostatitis/Chronic pelvic pain syndrome: An update on current clinical evidence. Rev. Urol. 14, 56–64. 10.3909/riu0557 23526487 PMC3602728

[B115] Nickel J. C. Xiang J. (2008). Clinical significance of nontraditional bacterial uropathogens in the management of chronic prostatitis. J. Urol. 179, 1391–1395. 10.1016/j.juro.2007.11.081 18289570

[B116] Nickel J. C. Olson M. E. Costerton J. W. (1991). Rat model of experimental bacterial prostatitis. Infection 19 Suppl 3, S126–S130. 10.1007/bf01643681 2055647

[B117] Nickel J. C. Downey J. Clark J. Ceri H. Olson M. (1995). Antibiotic pharmacokinetics in the inflamed prostate. J. Urol. 153, 527–529. 10.1097/00005392-199502000-00076 7815638

[B118] Nickel J. C. Shoskes D. Wang Y. Alexander R. B. Fowler J. J. Zeitlin S. (2006). How does the pre-massage and post-massage 2-glass test compare to the Meares-Stamey 4-glass test in men with chronic prostatitis/chronic pelvic pain syndrome? J. Urol. 176, 119–124. 10.1016/S0022-5347(06)00498-8 16753385

[B119] Nishikawa H. Yasuda M. Uchiyama J. Rashel M. Maeda Y. Takemura I. (2008). T-even-related bacteriophages as candidates for treatment of Escherichia coli urinary tract infections. Arch. Virol. 153, 507–515. 10.1007/s00705-007-0031-4 18188500

[B120] Novikov A. I. Zaezzhalkin V. V. Kucherov V. A. Frolov S. (2010). Immunocorrecting therapy of chronic bacterial prostatitis. Urologiia 44, 46–48. 20973134

[B121] Paterson D. L. Bonomo R. A. (2005). Extended-spectrum beta-lactamases: A clinical update. Clin. Microbiol. Rev. 18, 657–686. 10.1128/CMR.18.4.657-686.2005 16223952 PMC1265908

[B122] Peleg A. Y. Hooper D. C. (2010). Hospital-acquired infections due to gram-negative bacteria. N. Engl. J. Med. 362, 1804–1813. 10.1056/NEJMra0904124 20463340 PMC3107499

[B123] Perletti G. Skerk V. Magri V. Markotic A. Mazzoli S. Parnham M. J. (2011). Macrolides for the treatment of chronic bacterial prostatitis: An effective application of their unique pharmacokinetic and pharmacodynamic profile (Review). Mol. Med. Rep. 4, 1035–1044. 10.3892/mmr.2011.575 21874250

[B124] Perletti G. Marras E. Wagenlehner F. M. Magri V. (2013). Antimicrobial therapy for chronic bacterial prostatitis. Cochrane Database Syst. Rev. 8, D9071. 10.1002/14651858.CD009071.pub2 PMC11361477 23934982

[B125] Poirel L. Potron A. Nordmann P. (2012). OXA-48-like carbapenemases: The phantom menace. J. Antimicrob. Chemother. 67, 1597–1606. 10.1093/jac/dks121 22499996

[B126] Pontari M. A. (2002). Inflammation and anti-inflammatory therapy in chronic prostatitis. Urology 60, 29–33. 10.1016/s0090-4295(02)02381-6. 33-34. 12521589

[B127] Pontari M. A. Joyce G. F. Wise M. McNaughton-Collins M. (2007). Prostatitis. J. Urol. 177, 2050–2057. 10.1016/j.juro.2007.01.128 17509285

[B128] Pullukcu H. Tasbakan M. Sipahi O. R. Yamazhan T. Aydemir S. Ulusoy S. (2007). Fosfomycin in the treatment of extended spectrum beta-lactamase-producing Escherichia coli-related lower urinary tract infections. Int. J. Antimicrob. Agents 29, 62–65. 10.1016/j.ijantimicag.2006.08.039 17189097

[B129] Ramakrishnan K. Salinas R. C. (2010). Prostatitis: Acute and chronic. Primary Care 37, 547–563. 10.1016/j.pop.2010.04.007 20705198

[B130] Rees J. Abrahams M. Doble A. Cooper A. (2015). Diagnosis and treatment of chronic bacterial prostatitis and chronic prostatitis/chronic pelvic pain syndrome: A consensus guideline. BJU Int. 116, 509–525. 10.1111/bju.13101 25711488 PMC5008168

[B131] Rhodes N. J. Gardiner B. J. Neely M. N. Grayson M. L. Ellis A. G. Lawrentschuk N. (2015). Optimal timing of oral fosfomycin administration for pre-prostate biopsy prophylaxis. J. Antimicrob. Chemother. 70, 2068–2073. 10.1093/jac/dkv067 25802286

[B132] Roberts R. O. Lieber M. M. Rhodes T. Girman C. J. Bostwick D. G. Jacobsen S. J. (1998). Prevalence of a physician-assigned diagnosis of prostatitis: The Olmsted County Study of Urinary Symptoms and Health Status Among Men. Urology 51, 578–584. 10.1016/s0090-4295(98)00034-x 9586610

[B133] Rogers B. A. Ingram P. R. Runnegar N. Pitman M. C. Freeman J. T. Athan E. (2014). Community-onset Escherichia coli infection resistant to expanded-spectrum cephalosporins in low-prevalence countries. Antimicrob. Agents Chemother. 58, 2126–2134. 10.1128/AAC.02052-13 24468775 PMC4023745

[B134] Sastry S. Doi Y. (2016). Fosfomycin: Resurgence of an old companion. J. Infect. Chemother. 22, 273–280. 10.1016/j.jiac.2016.01.010 26923259 PMC4833629

[B135] Schaeffer A. J. (2006). Clinical practice. Chronic prostatitis and the chronic pelvic pain syndrome. N. Engl. J. Med. 355, 1690–1698. 10.1056/NEJMcp060423 17050893

[B136] Schoeb D. S. Schlager D. Boeker M. Wetterauer U. Schoenthaler M. Hermann T. R. W. (2017). Surgical therapy of prostatitis: A systematic review. World J. Urol. 35, 1659–1668. 10.1007/s00345-017-2054-0 28612108

[B137] Senol S. Tasbakan M. Pullukcu H. Sipahi O. R. Sipahi H. Yamazhan T. (2010). Carbapenem versus fosfomycin tromethanol in the treatment of Extended-Spectrum Beta-Lactamase-Producing escherichia Coli-Related complicated lower urinary tract infection. J. Chemother. 22, 355–357. 10.1179/joc.2010.22.5.355 21123160

[B138] Seo S. I. Lee S. J. Kim J. C. Choi Y. J. SW S. W. Hwang T. K. (2003). Effects of androgen deprivation on chronic bacterial prostatitis in a rat model. Int. J. Urol. 10, 485–491. 10.1046/j.1442-2042.2003.00666.x 12941127

[B139] Shang Y. Liu C. Cui D. Han G. Yi S. (2014). The effect of chronic bacterial prostatitis on semen quality in adult men: A meta-analysis of case-control studies. Sci. Rep. 4, 7233. 10.1038/srep07233 25429735 PMC4246207

[B140] Sharp V. J. Takacs E. B. Powell C. R. (2010). Prostatitis: Diagnosis and treatment. Am. Fam. Physician 82, 397–406. 10.1136/bmj.c3929 20704171

[B141] Shigehara K. Miyagi T. Nakashima T. Shimamura M. (2008). Acute bacterial prostatitis after transrectal prostate needle biopsy: Clinical analysis. J. Infect. Chemother. 14, 40–43. 10.1007/s10156-007-0570-3 18297448

[B142] Shoskes D. A. Zeitlin S. I. Shahed A. Rajfer J. (1999). Quercetin in men with category III chronic prostatitis: A preliminary prospective, double-blind, placebo-controlled trial. Urology 54, 960–963. 10.1016/s0090-4295(99)00358-1 10604689

[B143] Shrestha N. K. Amuh D. Goldman M. P. Riebel W. J. Tomford W. J. (2000). Treatment of a complicated Vancomycin-Resistant enterococcal urinary tract infection with fosfomycin. Infect. Dis. Clin. Pract. 9, 368–371. 10.1097/00019048-200009090-00004

[B144] Skerk V. Krhen I. Lisic M. Begovac J. Roglic S. Skerk V. (2004). Comparative randomized pilot study of azithromycin and doxycycline efficacy in the treatment of prostate infection caused by Chlamydia trachomatis. Int. J. Antimicrob. Agents 24, 188–191. 10.1016/j.ijantimicag.2004.03.014 15288321

[B145] Sohn D. W. Han C. H. Jung Y. S. Kim S. I. Kim S. W. Cho Y. H. (2009). Anti-inflammatory and antimicrobial effects of garlic and synergistic effect between garlic and ciprofloxacin in a chronic bacterial prostatitis rat model. Int. J. Antimicrob. Agents 34, 215–219. 10.1016/j.ijantimicag.2009.02.012 19375896

[B146] Sorgel F. Bulitta J. Kinzig-Schippers M. (2001). How well do gyrase inhibitors work? The pharmacokinetics of quinolones. Pharm. Unserer Zeit 30, 418–427. 10.1002/1615-1003(200109)30:5<418::AID-PAUZ418>3.0.CO;2- 11575179

[B147] Soto S. M. Smithson A. Martinez J. A. Horcajada J. P. Mensa J. Villa J. (2007). Biofilm formation in uropathogenic Escherichia coli strains: Relationship with prostatitis, urovirulence factors and antimicrobial resistance. J. Urol. 177, 365–368. 10.1016/j.juro.2006.08.081 17162092

[B148] Stamatiou K. Pierris N. (2017). Mounting resistance of uropathogens to antimicrobial agents: A retrospective study in patients with chronic bacterial prostatitis relapse. Invest. Clin. Urol. 58, 271–280. 10.4111/icu.2017.58.4.271 PMC5494352 28681038

[B149] Stoica G. Cariou G. Colau A. Cortesse A. Hoffmann P. Schaetz A. (2007). Epidemiology and treatment of acute prostatitis after prostatic biopsy. Prog. Urol. 17, 960–963. 10.1016/S1166-7087(07)92397-0 17969797

[B150] Tetz G. Tetz V. (2016). Bacteriophage infections of microbiota can lead to leaky gut in an experimental rodent model. Gut. Pathog. 8, 33. 10.1186/s13099-016-0109-1 27340433 PMC4918031

[B151] Van Belleghem J. D. Clement F. Merabishvili M. Lavigne R. Vaneechoutte M. (2017). Pro- and anti-inflammatory responses of peripheral blood mononuclear cells induced by Staphylococcus aureus and Pseudomonas aeruginosa phages. Sci. Rep. 7, 8004. 10.1038/s41598-017-08336-9 28808331 PMC5556114

[B152] Van Boeckel T. P. Gandra S. Ashok A. Caudron Q. Grenfell B. T. Levin S. A. (2014). Global antibiotic consumption 2000 to 2010: An analysis of national pharmaceutical sales data. Lancet Infect. Dis. 14, 742–750. 10.1016/S1473-3099(14)70780-7 25022435

[B153] Videcnik Z. J. Maticic M. Jeverica S. Smrkolj T. (2015). Diagnosis and treatment of bacterial prostatitis. Acta Dermatovenerol. Alp Pannonica Adriat 24, 25–29. 10.15570/actaapa.2015.8 26086164

[B154] Vidlar A. Vostalova J. Ulrichova J. Student V. Stejskal D. Reichenbach R. (2010). The effectiveness of dried cranberries (Vaccinium macrocarpon) in men with lower urinary tract symptoms. Br. J. Nutr. 104, 1181–1189. 10.1017/S0007114510002059 20804630

[B155] Vyas J. B. Ganpule S. A. Ganpule A. P. Sabnis R. B. Desai M. R. (2013). Transrectal ultrasound-guided aspiration in the management of prostatic abscess: A single-center experience. Indian J. Radiol. Imaging 23, 253–257. 10.4103/0971-3026.120262 24347857 PMC3843335

[B156] Wagenlehner F. M. Weidner W. Sorgel F. Naber K. G. (2005). The role of antibiotics in chronic bacterial prostatitis. Int. J. Antimicrob. Agents 26, 1–7. 10.1016/j.ijantimicag.2005.04.013 15970433

[B157] Wagenlehner F. M. Lunz J. C. Kees F. Wieland W. Naber K. G. (2006). Serum and prostatic tissue concentrations of moxifloxacin in patients undergoing transurethral resection of the prostate. J. Chemother. 18, 485–489. 10.1179/joc.2006.18.5.485 17127224

[B158] Wagenlehner F. M. Niemetz A. H. Weidner W. Naber K. G. (2008). Spectrum and antibiotic resistance of uropathogens from hospitalised patients with urinary tract infections: 1994-2005. Int. J. Antimicrob. Agents 31 Suppl 1, S25–S34. 10.1016/j.ijantimicag.2007.07.029 17997282

[B159] Wagenlehner F. M. Pilatz A. Bschleipfer T. Diemer T. Linn T. Meinhardt A. (2013). Bacterial prostatitis. World J. Urol. 31, 711–716. 10.1007/s00345-013-1055-x 23519458

[B160] Wailan A. M. Paterson D. L. (2014). The spread and acquisition of NDM-1: A multifactorial problem. Expert Rev. Anti Infect. Ther. 12, 91–115. 10.1586/14787210.2014.856756 24308710

[B161] Weidner W. Ludwig M. Brahler E. Schiefer H. G. (1999). Outcome of antibiotic therapy with ciprofloxacin in chronic bacterial prostatitis. Drugs 58 Suppl 2, 103–106. 10.2165/00003495-199958002-00021 10553717

[B162] Weinbauer M. G. (2004). Ecology of prokaryotic viruses. FEMS Microbiol. Rev. 28, 127–181. 10.1016/j.femsre.2003.08.001 15109783

[B163] Wise G. J. Shteynshlyuger A. (2008). Atypical infections of the prostate. Curr. Prostate Rep. 6, 86–93. 10.1007/s11918-008-0014-2

[B164] Wu X. R. Sun T. T. Medina J. J. (1996). In vitro binding of type 1-fimbriated Escherichia coli to uroplakins Ia and Ib: Relation to urinary tract infections. Proc. Natl. Acad. Sci. U. States America 93, 9630–9635. 10.1073/pnas.93.18.9630 PMC38479 8790381

[B165] Yazawa S. Nagata H. Kanao K. Kikuchi E. Hosokawa N. Shu H. (2013). Novel algorithm for predicting severe cases of acute bacterial prostatitis. J. Urol. 189, e475–e476. 10.1016/j.juro.2013.02.802

[B166] Yi S. Han G. Shang Y. Liu C. Cui D. Yu S. (2016). Microbubble-mediated ultrasound promotes accumulation of bone marrow mesenchymal stem cell to the prostate for treating chronic bacterial prostatitis in rats. Sci. Rep. 6, 19745. 10.1038/srep19745 26797392 PMC4726435

[B167] Yoon B. I. Ha U. S. Sohn D. W. Lee S. J. Kim H. W. Han C. H. (2011). Anti-inflammatory and antimicrobial effects of nanocatechin in a chronic bacterial prostatitis rat model. J. Infect. Chemother. 17, 189–194. 10.1007/s10156-010-0098-9 20694569

[B168] Yoon B. I. Kim S. Han D. S. Ha U. S. Lee S. J. Kim H. W. (2012). Acute bacterial prostatitis: How to prevent and manage chronic infection? J. Infect. Chemother. 18, 444–450. 10.1007/s10156-011-0350-y 22215226

[B169] Yoon B. I. Bae W. J. Choi Y. S. Kim S. J. Ha U. S. Hong S. H. (2018). Anti-inflammatory and Antimicrobial Effects of Anthocyanin Extracted from Black Soybean on Chronic Bacterial Prostatitis Rat Model. Chin. J. Integr. Med. 24, 621–626. 10.1007/s11655-013-1547-y 24126975

[B170] Yosef I. Manor M. Kiro R. Qimron U. (2015). Temperate and lytic bacteriophages programmed to sensitize and kill antibiotic-resistant bacteria. Proc. Natl. Acad. Sci. U. S. A 112, 7267–7272. 10.1073/pnas.1500107112 26060300 PMC4466736

[B171] Zalmanovici T. A. Green H. Paul M. Yaphe J. Leibovici L. (2010). Antimicrobial agents for treating uncomplicated urinary tract infection in women. Cochrane Database Syst. Rev. 10, D7182. 10.1002/14651858.CD007182.pub2 PMC12501835 20927755

[B172] Zegarra M. L. Sanchez M. A. Loza M. C. Gutierrez E. C. (2008). Semen and urine culture in the diagnosis of chronic bacterial prostatitis. Int. Braz. J. Urol. 34 30-37, 38–40. 10.1590/s1677-55382008000100006 18341719

[B173] Zhanel G. G. Walkty A. J. Karlowsky J. A. (2016). Fosfomycin: A First-Line oral therapy for acute uncomplicated cystitis. Can. J. Infect. Dis. Med. Microbiol. 2016, 2082693. 10.1155/2016/2082693 27366158 PMC4904571

[B174] Zhanel G. G. Zhanel M. A. Karlowsky J. A. (2018). Oral fosfomycin for the treatment of acute and chronic bacterial prostatitis caused by Multidrug-Resistant escherichia coli. Can. J. Infect. Dis. Med. Microbiol. 2018, 1404813. 10.1155/2018/1404813 29666664 PMC5831921

[B175] Zhang Z. C. Jin F. S. Liu D. M. Shen Z. J. Sun Y. H. Guo Y. L. (2012). Safety and efficacy of levofloxacin versus ciprofloxacin for the treatment of chronic bacterial prostatitis in Chinese patients. Asian J. Androl. 14, 870–874. 10.1038/aja.2012.48 22864282 PMC3720113

[B176] Zhao W. P. Li Y. T. Chen J. Zhang Z. G. Jiang H. Xia D. (2012). Prostatic calculi influence the antimicrobial efficacy in men with chronic bacterial prostatitis. Asian J. Androl. 14, 715–719. 10.1038/aja.2012.40 22796735 PMC3734994

[B177] Zhou J. F. Xiao W. Q. Zheng Y. C. Dong J. Zhang S. M. (2006). Increased oxidative stress and oxidative damage associated with chronic bacterial prostatitis. Asian J. Androl. 8, 317–323. 10.1111/j.1745-7262.2006.00144.x 16625281

[B178] Zowawi H. M. Harris P. N. Roberts M. J. Tambyah P. A. Schembri M. A. Pezzani M. D. (2015). The emerging threat of multidrug-resistant Gram-negative bacteria in urology. Nat. Rev. Urol. 12, 570–584. 10.1038/nrurol.2015.199 26334085

